# The deacetylase NagA mediates the remodeling and recycling of peptidoglycan-derived amino sugars in mycobacteria

**DOI:** 10.1016/j.jbc.2025.110597

**Published:** 2025-08-14

**Authors:** Collette S. Guy, Charlotte Cooper, Magdalena Karlikowska, James Harrison, Albel Singh, Luis Steven Servín-González, Caroline A. Evans, Saskia E. Bakker, Andrew Bottrill, Apoorva Bhatt, Stéphane Mesnage, Gurdyal S. Besra, Elizabeth Fullam

**Affiliations:** 1 School of Life Sciences, University of Warwick, Coventry, UK; 2 Institute of Microbiology & Infection, School of Biosciences, University of Birmingham, Birmingham, UK; 3 Department of Chemical and Biological Engineering, ChELSI Institute, University of Sheffield, Sheffield, UK; 4 School of Biosciences, University of Sheffield, Sheffield, UK; 5 Manchester Institute of Biotechnology, University of Manchester, Manchester, UK; 6 Department of Chemistry, University of Manchester, Manchester, UK

**Keywords:** mycobacteria, peptidoglycan, *Mycobacterium tuberculosis*, NagA, cell wall, Nacetylglucosamine, N-acetylglucosamine-6-phosphate deacetylase, metabolism, biofilm, amino sugar, cell wall recycling, transport

## Abstract

Many bacterial species are known to recover peptidoglycan (PG) fragments released from remodeling of their cell walls during growth and cell division. These PG fragments not only provide an essential energy resource, especially in nutrient restricted environments, but also play a critical role in influencing infection. Yet whether mycobacteria have the capacity to recycle their PG, or not, has still not been resolved. In this study, we show that NagA, an *N*-acetylglucosamine-6-phosphate (GlcNAc-6-P) deacetylase, is essential for coordinating the remodeling and recycling of an amino sugar component released from the mycobacterial cell wall. We show that NagA is exclusively responsible for GlcNAc-6-P deacetylation and is pivotal for the *de novo* synthesis of core cell wall building blocks. Indeed, a *nagA* deletion mutant exhibited an altered composition of the cell envelope, smaller overall cell size, defective biofilm formation, and enhanced susceptibility to cell wall targeting agents. Moreover, uptake analysis and profiling of the amino sugar pool revealed that NagA inactivation blocks *N*-acetylglucosamine (GlcNAc) import and has a pronounced effect on the fate and levels of the intracellular amino sugar pool. Loss of NagA led to the upregulation and downregulation of proteins involved in cell wall biosynthesis, thereby altering cell wall homeostasis. Overall, our data highlight the importance of an overlooked yet conserved component in an important PG salvage pathway in mycobacteria, in which NagA provides a unique GlcNAc sensing mechanism, thus acting as a checkpoint for regulating the recovery and reuse of PG fragments.

*Mycobacterium tuberculosis* (*Mtb*), the causative agent of tuberculosis (TB) is, arguably, one of the world’s most successful pathogens globally, responsible for over ∼1 billion deaths over the last 2000 years (1). The World Health Organization estimates that in 2023 there were ∼10.8 million new active TB cases and 1.3 million deaths resulting from TB infection, placing *Mtb* as one of the leading causes of death from a single infectious agent worldwide (https://www.who.int/tb/publications/global_report/en/). Although there are effective treatment regimens against drug-susceptible TB, poor compliance and the lack of new therapeutic options have led to the emergence and escalation of not only drug-resistant, but also untreatable strains of *Mtb*, which is jeopardizing efforts to control the TB epidemic (2). Clearly, there is an urgent need to identify alternative pathways that can be targeted with novel treatment strategies to combat this major global health challenge.

One of the distinguishing features of the *Mtb* pathogen is a highly unique cell envelope, which is integral to its virulence and survival (3, 4, 5). Given its essentiality, cell wall synthetic pathways have been exploited as targets of many current first*-* and second-line TB therapeutics, as well as those under development in the drug development pipeline. Yet despite this vulnerability, not all the pathways involved in cell wall synthesis have been explored or targeted. The mycobacterial cell wall is a complex macromolecular structure comprising of an interconnected peptidoglycan, arabinogalactan and long chain mycolic acid (mAGP) core, interspersed with additional ”free” lipids and glycolipids that form an outer “myco-membrane” and an outer capsule composed predominantly of an α-glucan polysaccharide (3, 4, 5). The inner peptidoglycan (PG) mesh acts as an attachment scaffold for arabinogalactan (AG) and has a substantial role in the structural integrity and tensile strength of the cell and protection against osmotic pressure. In mycobacteria, PG consists of glycan strands of an alternating β(1 → 4) linked *N*-acetylglucosamine (GlcNAc) to either an *N*-acetylmuramic acid (MurNAc) or an *N*-glycolyl derivative (MurNGly), distinguishing it from other bacterial species, with adjacent chains cross-linked through short peptide chains (6, 7). Further modifications are *via* a unique α-1-rhamnopyranose-(1 → 3)-α−D-GlcNAc-(1→P) linker unit, which attaches approximately 10 to 12% of the muramic acid residues of PG to AG (8).

In mycobacteria the biosynthesis of PG and the unit linking PG to AG both require the UDP-GlcNAc building block, the *de novo* synthesis of which relies on the supply of the glucosamine-6-phosphate (GlcN-6-P) precursor, which sits at the crossroads of the glycolysis and cell wall biosynthetic pathways (Fig. 1) (9). GlcN-6-P is generated either from the glycolysis pathway, where GlmS catalyzes the isomerization of fructose-6-phosphate to GlcN-6-P (10, 11), or alternatively GlcN-6-P can be derived from the deacetylation of GlcNAc-6-P by NagA (12, 13). Because there are no obvious GlcN-6-P acetyltransferases in mycobacteria (14), the most likely origin of cytosolic GlcNAc-6-P is through the recycling, import and phosphorylation of GlcNAc released from remodeled PG. GlcN-6-P is subsequently converted to UDP-GlcNAc in three steps *via* the formation of glucosamine-1-phosphate (GlcN-1-P) and the *N-*acetyl-glucosamine-1-phosphate (GlcNAc-1-P) intermediate (Fig. 1) by the sequential action of GlmM and GlmU (15, 16, 17). The UDP-GlcNAc building block is then utilized to form the cell wall.

**Figure 1:**
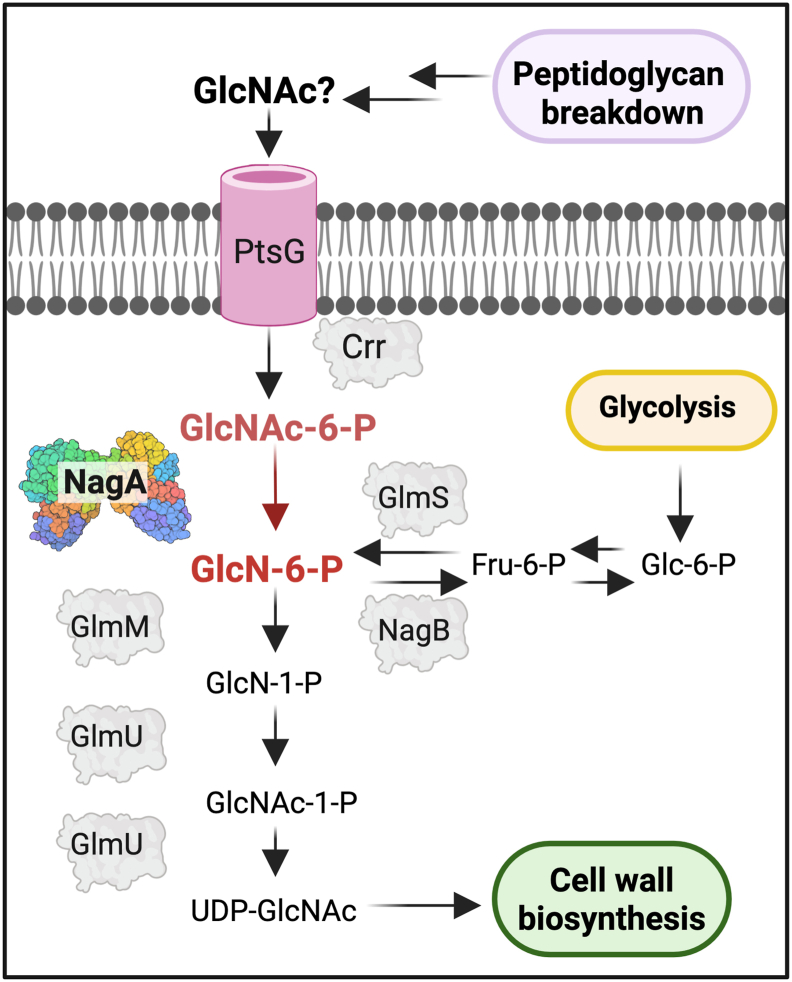
**Overview of the GlcNAc recycling pathway in *Mycobacterium. smegmatis*.** In *M. smegmatis* GlcNAc is likely imported by PtsG and phosphorylated by Crr to form GlcNAc-6-P and further deacetylated by NagA to glucosamine 6-phosphate (GlcN-6-P). The pathway then diverges, and GlcN-6-P is either shunted into cell wall biosynthesis or converted to fructose 6-phosphate (Fru-6-P) by the action of NagB to enter the glycolysis pathway. Created with BioRender.

In various bacterial species, the NagA pathway has been shown to have an important role in a plethora of processes that range from regulating GlcNAc assimilation and metabolism; triggering cell signaling pathways, perturbing the intracellular amino sugar pool; maintaining cell wall biosynthesis and homeostasis, coordinating PG recycling and, in *Streptomyces*, controlling antibiotic production (18, 19, 20, 21, 22, 23, 24, 25, 26). The exogenous supply of GlcNAc is likely derived predominantly from core bacterial cell wall components, and pathways to recover GlcNAc and other PG fragments to ensure scarce resources are not otherwise lost are well established in many bacterial species. However, the mechanisms that mycobacteria deploy to recycle their cell walls are less clear, despite mycobacterial PG metabolites enabling key aspects of *Mtb* biology, such as resuscitation, virulence, cell division, and cell wall synthesis (27, 28, 29, 30). Although the complete repertoire of mycobacteria cell wall recycling enzymes has not yet been identified, an exception is NagA. We have previously shown that the mycobacterial NagA enzyme is responsible, and selective, for the deacetylation of GlcNAc-6-P (12). But very little is known about the role of NagA in mycobacterial cells; however, proteomic profiling identified NagA is present in lung tissues from guinea pigs infected with *Mtb*, pointing toward a potential role in *Mtb* during infection (31). In addition, *nagA* is highly conserved across mycobacterial species, including *Mycobacterium leprae*, an obligate pathogen that has undergone extensive gene decay resulting in a core set of genes considered essential to facilitate intracellular survival in humans (Fig. S1) (32), providing evidence that the NagA pathway is also an important metabolic process for mycobacteria.

In this study, we sought to investigate the role of the previously overlooked NagA enzyme in *Mycobacterium smegmatis*. We demonstrate that NagA is indeed exclusively responsible for the GlcNAc-6-P deacetylase activity in mycobacterial cells and provide evidence linking its function to GlcNAc uptake and regulation of intracellular levels of the amino sugar pool. Loss of NagA activity resulted in defective PG biosynthesis leading to increased susceptibility to PG targeting agents, smaller cells, and impaired biofilm formation, underscoring its role in maintaining cell wall homeostasis. Overall, our data indicate that NagA is a key player in recycling remodeled PG fragments in mycobacteria, contributing to the control and use of scarce nutrient resources during infection.

## Results

### NagA is required for the deacetylation of GlcNAc-6-P

To assess the importance of GlcNAc-6-P deacetylase activity in mycobacteria, we constructed an in-frame deletion mutant of *nagA* in *M. smegmatis*, which was confirmed by whole genome sequencing (Fig. S2) and expression of the adjacent genes from extracted RNA (Fig. S2). To validate NagA function in *M. smegmatis*, we monitored GlcNAc-6-P deacetylase activity in cell lysates, which fell from 32.9 nmol/min/mg of protein in the WT strain to undetectable levels in the mutant. This activity is consistent with our prior biochemical data (12), indicating that under the conditions tested NagA is the only enzyme responsible for the deacetylation of GlcNAc-6-P in mycobacterial cells and establishes that this activity is not functionally compensated by other pathways, despite the high level of genetic redundancy in *M. smegmatis*.

### NagA is essential for utilizing GlcNAc as a sole carbon source

**Figure 2:**
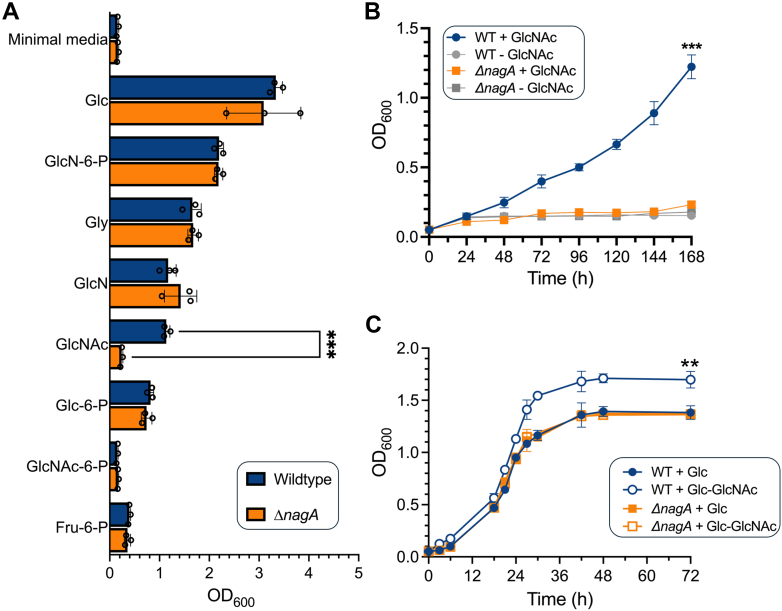
**GlcNAc availability and use by NagA influences mycobacterial growth.***A*, growth at 7 days of WT (*blue*) and Δ*nagA* (*orange*) in minimal media supplemented with 20 mM glucose (Glc), glucosamine 6-phosphate (GlcN-6-P), glycerol (Gly), glucosamine (GlcN), *N*-acetyl glucosamine (GlcNAc), glucose 6-phosphate (Glc-6-P), glucosamine 6-phosphate (GlcN-6-P), *N*-acetyl glucosamine 6-phosphate (GlcNAc-6-P), fructose 6-phosphate (Fru-6-P), *B*, growth curves of WT (*blue*) and Δ*nagA* (*orange*) in minimal media in the presence or absence of 20 mM GlcNAc. *C*, growth curves of WT (*blue*) and Δ*nagA* (*orange*) in minimal media supplemented with either 5 mM glucose or 5 mM glucose and 1 mM GlcNAc. Error bars represent standard deviation from three biological replicates. Statistical significance was determined using unpaired *t-*tests ∗ = *p* < 0.05, ∗∗ = *p* < 0.01 ∗∗∗ = *p* < 0.001.

To determine the impact of *nagA* deletion on the ability of *M. smegmatis* to utilize various carbon sources, strains were starved and growth assessed after 7 days in minimal media supplemented with glycerol (Gly), glucose (Glc), glucose 6-phosphate (Glc-6-P), fructose 6-phosphate (Fru-6-P), glucosamine (GlcN), GlcN-6-P, GlcNAc, and GlcNAc-6-P. Wild type (WT) *M. smegmatis* preferentially utilized Glc > GlcN-6-P > Gly > GlcN > GlcNAc > Glc-6-P as a sole carbon source but did not grow on GlcNAc-6-P or Fru-6-P supplemented media (Fig. 2*A*). The pattern of carbon source utilization for Δ*nagA* mirrored the parental strain except for GlcNAc, where the WT strain grew but Δ*nagA* did not. To analyze if the growth defect occurred during all growth phases, we monitored the absorbance during the lag phase and transition to exponential growth and found Δ*nagA* still failed to grow (Fig. 2*B*). Next, to examine whether NagA inactivation causes bacteriostasis or lethality in *M. smegmatis*, we monitored the ability to form colony forming units (CFUs). No significant differences in the viability of both strains were observed following nutrient starvation or growth in minimal media over the experimental time course (Fig. S3). As expected, the CFUs increased for the WT strain supplemented with GlcNAc but not for Δ*nagA*, which remained viable but static (Fig. S3), suggesting that under these low nutrient conditions an impaired ability to use GlcNAc by the Δ*nagA* mutant is responsible for the observed phenotype. When supplied with glucose, its preferred carbon source, and GlcNAc, a dual carbon condition that promotes the induction of *nagA* and incorporation of exogenous GlcNAc into PG (33), we found enhanced growth of the WT strain (Fig. 2*C*). In contrast the *nagA* mutant consistently reached lower biomass levels under these conditions, with a growth phenotype that closely resembles that of cells grown with glucose alone (Fig. 2*C*). Taken together, this strongly suggests that NagA has a pivotal role in GlcNAc utilization and controlling mycobacterial growth in response to exogenous GlcNAc availability.

### NagA controls GlcNAc uptake and the incorporation of exogenous GlcNAc into the cell wall

**Figure 3:**
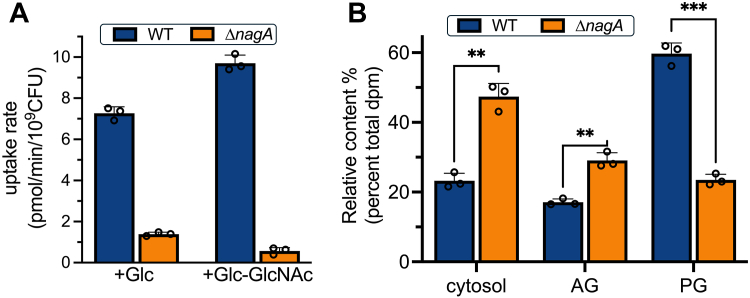
**Deletion of *nagA* prevents^14^C-GlcNAc uptake and alters incorporation of exogenous ^14^C-GlcNAc into cell walls.***A*, GlcNAc uptake rates in wild type and Δ*nagA* strains grown in minimal media supplemented with either 5 mM glucose or 5 mM glucose and 1 mM GlcNAc; *B*, incorporation of ^14^C-GlcNAc into the cytosol, arabinogalactan (AG), and peptidoglycan (PG) of *Mycobacterium smegmatis.* WT and Δ*nagA* grown in minimal media with 5 mM glucose and 1 mM GlcNAc. Error bars represent standard deviation from three biological replicates. Statistical significance was determined using unpaired *t-*tests ∗ = *p* < 0.05, ∗∗ = *p* < 0.01 ∗∗∗ = *p* < 0.001.

Because our growth phenotypes indicate NagA controls how *M. smegmatis* accesses GlcNAc, we wanted to determine whether inactivation of GlcNAc-6-P deacetylase activity influences the entry of this amino sugar into the cell. To test this, we performed uptake assays with ^14^C-GlcNAc in mid-log phase WT and Δ*nagA* cells. Radiolabeled GlcNAc was rapidly taken up in WT with a rate of 7.3 ± 0.3 pmol/min/10^9^ CFU, whereas much lower levels of this amino sugar accumulated in the *nagA* mutant, and its uptake rate was much slower (1.4 ± 0.1 pmol/min/10^9^ CFU) (Fig. 3*A*). Cells grown in the presence of both Glc and GlcNAc displayed an ∼1.3-fold increase in uptake rate (9.7 ± 0.4 pmol/min/10^9^ CFU) in WT, whereas a ∼2.3-fold (0.6 ± 0.2 pmol/min/10^9^ CFU) reduction was observed in Δ*nagA* (Fig. 3*A*).

As GlcNAc is ultimately incorporated into the mycobacterial cell wall, predominantly as PG but also in the GlcNAc linker unit of AG, we then asked if impaired GlcNAc-6-P deacetylase activity disrupts the intracellular processing of this amino sugar building block. Following exposure to ^14^C-GlcNAc, we mapped the fate of the radiolabel by analyzing the incorporation of radioactivity into isolated cytosolic, PG and AG components from WT and the *nagA* mutant (Fig. 3*B*). As a proportion of the total cellular uptake, ^14^C-GlcNAc, or ^14^C-GlcNAc metabolite/s, accumulated at much higher levels in the cytosol of the *nagA* deletion mutant. Also, the Δ*nagA* strain showed a pronounced reduction in ^14^C-labeled PG. Surprisingly, AG extracted from Δ*nagA* showed a higher ratio of ^14^C-labeled AG to PG, indicating that loss of NagA activity leads to the UDP-GlcNAc cell wall precursor, common to both AG and PG pathways, being preferentially directed to form the linker unit attaching arabinan to PG. Collectively, this provides evidence that NagA acts a gatekeeper that regulates the recycling of PG derived amino sugars and maintains cell wall homeostasis.

### Deletion of *nagA* increases susceptibility to cell wall targeting agents

We reasoned that reduced incorporation of ^14^C-GlcNAc into the cell-envelope may lead to defects or alterations that modify its sensitivity to antibiotics and cell wall targeting agents. To probe this further, we assessed susceptibility of the *nagA* deletion strain against a panel of antibiotics and cell wall targeting agents by the resazurin microtitre assay (Table S1). We found that deletion of *nagA* resulted in increased susceptibility to the PG targeting agents vancomycin, cycloserine, and lysozyme; β-lactams in the presence of the β-lactamase inhibitor clavulanic acid; and the cell wall synthesis inhibitors isoniazid and ethambutol (Table S1 and S2). Spot assays confirmed the observed two-fold reductions in minimum inhibitory concentrations (MICs) with reduced recovery for the Δ*nagA* strain for each antibiotic, except ethambutol (Table S3). The discrepancy for ethambutol may be due to differences in its availability in agar *versus* liquid broth. No changes in the MICs for other antibiotic classes between the two strains were observed. To assess the integrity of the cell wall we tested the susceptibility of Δ*nagA* to lysis after incubation with lysozyme and found a significant reduction in survival of the *nagA* deletion mutant compared to the WT strain (Fig. S4*A*). This increased susceptibility is not due to a change in cell wall permeability as determined by ethidium bromide uptake (Fig. S4*B*). Additional lipid profiling revealed no qualitative differences of the Δ*nagA* mutant grown *in vitro* (Fig. S5), and comparable levels of Congo red staining for both strains (Fig. S4*C*) suggesting that the lipid layers of the cell wall are not altered upon *nagA* deletion. Collectively, these results demonstrate that deletion of *nagA* impairs the mycobacterial cell wall integrity, predominantly by altering PG formation.

### PG composition of mid-log phase *M. smegmatis* is altered by the deletion of NagA

Given the increased susceptibility of the *nagA* mutant to PG-targeting agents, we hypothesized that NagA activity influences the structure and composition of mycobacterial PG. To test this, WT and Δ*nagA* cells were cultured to mid-log and stationary phases with or without the addition of GlcNAc, and their extracted PG was analyzed using liquid chromatography-tandem mass spectrometry (LC-MS/MS). Across all samples, 29 PG monomers and 20 PG dimers were identified (Table 1, Fig. S6). For both strains the predominant muropeptide species were monomers (∼74–87%) (Table 1). In exponential growth the *nagA* mutant displayed an altered PG profile compared to WT. When grown in the presence of GlcNAc, the Δ*nagA* strain exhibited a higher dimer-to-monomer ratio (∼1:3) compared to the WT strain (∼1:6) indicating a higher extent of crosslinking in the mutant. In the absence of GlcNAc, the Δ*nagA* mutant peptidoglycan showed a higher abundance of glycolylated muramic acid (∼64% *versus* 46% for WT) along with a reduced abundance of acetylated muramic acid (23% *versus* 33% for WT) and deacetylated MurNAc (∼13% *versus* 22% of MurN for WT). These findings suggest that disruption of NagA alters the PG composition and polymerization, which may impact the integrity and physical properties of the cell envelope.

**Table 1:** Peptidoglycan composition of WT and Δ*nagA* strains

Muropeptide	18 h + GlcNAc		18 h - GlcNAc		48 h + GlcNAc		48 h - GlcNAc	
	WT	Δ*nagA*	WT	Δ*nagA*	WT	Δ*nagA*	WT	Δ*nagA*
% monomers	85.8	74.4	84.3	83.2	85.3	86.2	87.3	82.7
% dimers	14.2	25.6	15.7	16.8	14.7	13.8	12.7	17.3
Dimer:monomer ratio	1:6.0	1: 2.9	1:5.4	1:5.0	1:5.8	1:6.2	1:6.9	1:4.8
GlcNAc-MurGlyc (%)	93.3	98.6	45.7	64.2	99.4	99.6	98.6	98.6
GlcNAc-MurNAc (%)	6.3	0.5	32.6	22.9	0.4	0.2	0.7	0.5
GlcNAc-Mur (%)	0.4	1.0	21.8	12.9	0.2	0.1	0.8	0.7

The full list of monomers and dimers identified by LC-MS/MS are shown in Supplementary file S3.

GlcNAc, *N*-acetylglucosamine; MurNAc, *N*-acetylmuramic acid; MurGlyc, *N*-glycolylated muramic acid; Mur, muramic acid.

### Deletion of *nagA* results in shorter cells and altered cell wall thickness

**Figure 4:**
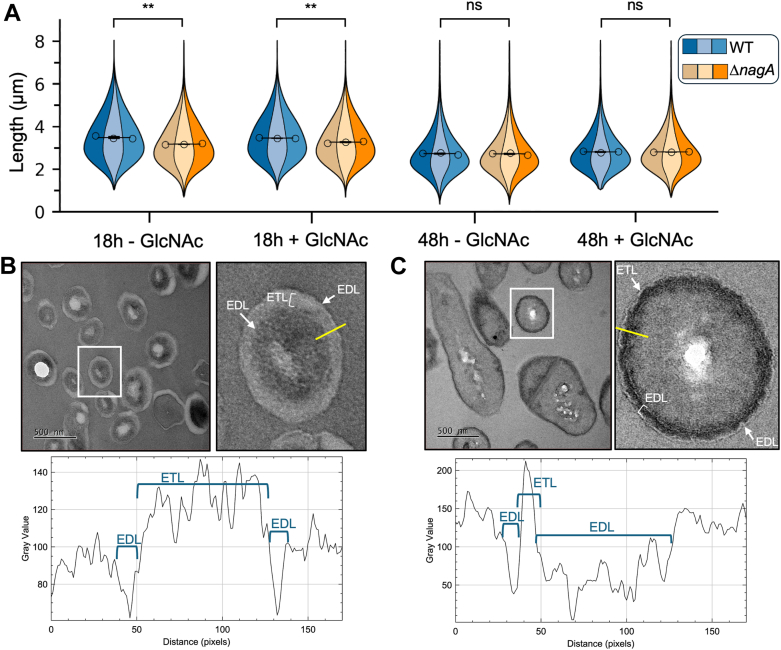
**NagA influences mycobacterial cell morphology.** WT and *ΔnagA* strains were grown in Sauton’s media supplemented with either glucose (5 mM) or glucose (5 mM) and GlcNAc (1 mM) and analyzed by imaging flow cytometry and TEM at 18 h and 48 h. *A*, cell length; error bars represent standard deviation from three biological replicates. Statistical significance was determined using unpaired *t-*tests ∗ = *p* < 0.05, ∗∗ = *p* < 0.01 ∗∗∗ = *p* < 0.001. Plots were made with the superviolin package in Python3.x Miniconda. *B*, TEM images of WT (18 h; Sauton’s minimal media supplemented with 5 mM glucose (5 mM) and GlcNAc (1 mM)). *C*, TEM images of Δ*nagA* (18 h; Sauton’s minimal media supplemented with 5 mM glucose (5 mM), and GlcNAc (1 mM) GlcNAc. The white square indicates the cell zoomed in on. Gray scale intensity plots measured across the *yellow dashed line*. Additional TEM images for WT and Δ*nagA* strains at 18 h and 48 h are shown in Figs. S7-S10. Fig. 4*B* is included in Fig. S8A. Fig. 4C is included in Fig. S8B. EDL, electron dense layer; ETL, electron translucent layer; TEM, transmission electron microscopy.

Since the PG composition of the Δ*nagA* strain differs from that of the WT and given the critical role of PG composition and architecture in maintaining bacterial cell shape (34) we investigated NagA’s role in defining mycobacterial cell morphology. WT and Δ*nagA* strains were cultured in Sauton’s minimal media supplemented with either glucose or glucose and GlcNAc and their morphology assessed during mid-log and stationary growth phases. Imaging cell flow cytometry revealed that Δ*nagA* cells were significantly shorter than WT cells in mid-log-phase, regardless of GlcNAc presence. (Fig. 4). In contrast, no differences in cell size were observed between the strains in stationary phase, when no cell division occurs (Fig. 4). To probe this result further, transmission electron microscopy (TEM) was used to examine the ultrastructure of the cell wall. As expected, WT cells exhibited normal morphology characterized by an inner electron dense layer (EDL) which corresponds to PG, an electron translucent layer representing the mycolyl-arabinogalactan and an outer EDL consisting of external lipids (35, 36, 37) (Figs. 4*B* and S7–S10). In contrast, as shown in Figure 4*C*, the Δ*nagA* the mutant strain exhibited notable morphological changes with a much thicker inner EDL and a reduced electron translucent layer, while the outer EDL remained similar to WT cells, consistent with our lipid analyses (Fig. S5). This indicates that *nagA* cells have a thicker PG layer and smaller AG region than WT cells. Together, these data point toward NagA’s involvement in controlling cell shape and morphology during cell-division.

### Deletion of *nagA* leads to the altered abundance of PG recycling and synthesis pathway metabolites

**Figure 5:**
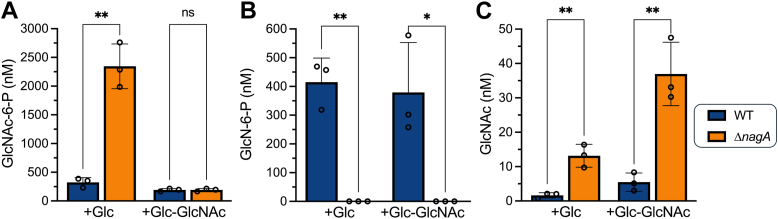
**NagA catalysis of GlcNAc-6-P is required to maintain the intracellular aminosugar pool.** High-performance anion-exchange chromatograph with pulsed amperometric detection (HPAEC-PAD) analysis of WT and Δ*nagA* cytosolic extracts of strains grown in minimal media supplemented with either 5 mM glucose or, 5 mM glucose and 1 mM GlcNAc. *A*, cytosolic GlcNAc-6-P concentration *B*, cytosolic GlcN-6-P concentration, *C*, cytosolic GlcNAc concentration. Error bars represent standard deviation from three biological replicates. Statistical significance was determined using unpaired *t-*tests ∗ = *p* < 0.05, ∗∗ = *p* < 0.01 ∗∗∗ = *p* < 0.001.

As we observed a pronounced reduction in GlcNAc uptake, alongside the substantial accumulation of ^14^C in the cytosol and altered cell wall in Δ*nagA*, we hypothesized that the intracellular amino sugar pool of the *nagA* mutant might be perturbed. To test this hypothesis, we quantified the amino sugars immediately upstream and downstream of NagA, in WT and mutant cytosolic extracts by ion chromatography with pulsed amperometric detection (Fig. 5). Inactivation of NagA led to an extensive build-up of GlcNAc-6-P (Fig. 5*A*), whereas the intracellular GlcN-6-P pool was completely depleted (Fig. 5*B*) even though this metabolite can also be produced by the glycolytic pathway. Although GlcNAc is thought to be internalized *via* a phosphoenolpyruvate-dependent sugar phosphotransferase system (PTS) in *M. smegmatis*, we unexpectedly observed a substantial accumulation of GlcNAc, rather than GlcNAc-6-P, in Δ*nagA* cells grown in the presence of exogenously supplied GlcNAc (Fig. 5*C*) suggesting additional routes for GlcNAc assimilation. Collectively, these findings establish that inactivation of NagA perturbs the intracellular reservoir and the generation of key amino sugar precursors thus regulating amino sugar flux.

### NagA deacetylase activity is required for biofilm formation

**Figure 6:**
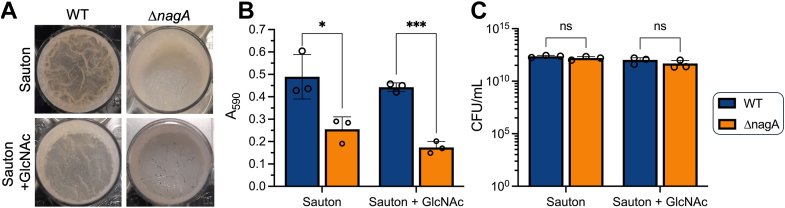
**The *nagA* deletion mutant exhibits a defect in biofilm formation.***A,* images of 7-day old WT and Δ*nagA* biofilms grown in detergent-free Sauton media in the presence or absence of 20 mM GlcNAc. *B,* crystal violet biofilm quantification. *C,* colony forming unit (CFU) enumeration of strains after 7 days of biofilm formation. Error bars represent SD from three biological replicates. Statistical significance was determined by unpaired *t* tests ∗ = *p* < 0.05, ∗∗ = *p* < 0.01, and ∗∗∗ = *p* < 0.001.

Because impaired PG biosynthesis is known to result in defective biofilms (38, 39), we speculated that blocking the NagA PG recycling pathway might also be key for biofilm formation. We assessed the formation of WT and Δ*nagA* pellicular biofilms formed at the air-liquid interface over 7 days. As shown in Figure 6, while biofilms of the *nagA* mutant strain could be observed, they exhibit a much smoother, more fragile morphology in contrast to the characteristic robust, wrinkled biofilms of WT. Next, we wanted to establish if elevated exogenous GlcNAc levels impacted on biofilm formation in both strains. We found that the WT strain switched biofilm phenotype, which now closely resembles the smooth morphology seen in the *nagA* mutant, while the biofilm phenotype of Δ*nagA* is further accentuated (Fig. 6*A*). Crystal violet quantification showed a significant reduction in the biofilm biomass of Δ*nagA* compared to WT under both conditions (Fig. 6*B*). In contrast, the total biomass amount for WT or Δ*nagA* was similar regardless of the presence or absence of GlcNAc (Fig. 6*B*). To determine whether the altered biofilm biomass is linked to viability, we assessed CFU counts and found no differences between the WT and *nagA* mutant, indicating that impaired biofilm formation is not a consequence of changes in bacterial growth rate or viability (Fig. 6*C*). These findings demonstrate the crucial importance of NagA activity for effective biofilm formation.

### Global proteomic analysis revealed NagA influences PG, AG, and capsular glucan pathways

**Figure 7:**
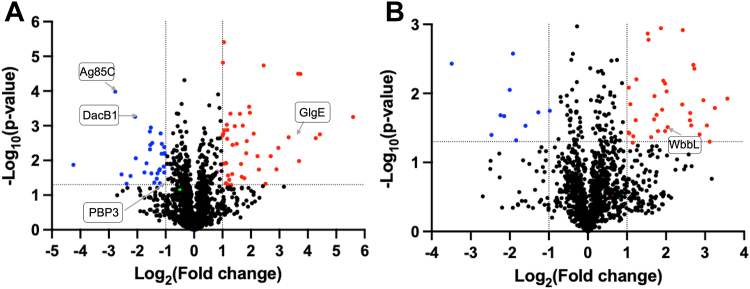
**Comparative proteomic analysis of *Mycobacterium smegmatis* WT and the *nagA* deletion mutant.** Volcano plots illustrating the differential protein abundance of (*A*) soluble and (*B*) membrane proteins between the WT and *nagA* deletion mutant strains. *Blue* corresponds to proteins with significant (*p* =< 0.05) < -1 log_2_ fold change abundance. *Red* corresponds to proteins with significant (*p* =< 0.05) >1 log_2_ fold change abundance. Proteins involved in cell wall synthesis pathways are highlighted. The full proteomics dataset is listed in Supplementary file S4.

Next, to examine the effect of *nagA* disruption on wider biosynthetic pathways, we performed whole cell proteomics of mid-exponential WT and Δ*nagA*. Differential expression analysis revealed that 76 proteins are found in higher abundance and 38 proteins in lower abundance in the *nagA* mutant based on a log2 fold change (FC) > ± 1 and adjusted *p* value ≤ 0.05) (Fig. 7, Supplementary File S4). Most notably, the *nagA* mutant showed pronounced changes in the abundance of specific enzymes in the PG and AG biosynthetic pathways. Specifically, we found over four-fold higher levels of WbbL1 (MSMEG_1826) in the *nagA* deletion mutant. WbbL1 is responsible for the formation of the AG-PG linker (40). This finding is consistent with our radiolabeling studies that showed Δ*nagA* preferentially funnels 14C-GlcNAc into AG rather than PG. In contrast, we observed a ∼4-fold reduction in the levels of the D-alanyl-D-alanine carboxypeptidase DacB2 (MSMEG_2433), involved in removing the terminal D-alanine residue from the pentapeptide sidechains (41), and a ∼2-fold reduction in levels of the probable penicillin binding protein transpeptidase Pbp3 (MSMEG_4233, Rv2163c). Examination of the proteins encoded in the *nagA* operon revealed unaltered levels of Crr (MSMEG_2117) and a significant 1.8-fold increase in abundance for NagB (MSMEG_2119). NagB funnels GlcN-6-P into the glycolysis pathway (Fig. 1), suggesting a mechanism to regulate the intersecting cell wall biosynthesis and glycolysis pathways in the absence of NagA. The Δ*nagA* strain also showed pronounced alterations of proteins levels involved in the biosynthesis of other mycobacterial cell envelope constituents. The abundance of GlgE, (MSMEG_4916), involved in the glucan capsule synthesis (42) increased ∼10-fold, whereas Ag85C (MSMEG_6583), responsible for trehalose mycolate synthesis (43) showed a significant decrease of ∼7-fold. Combined, our findings further point toward a global role for NagA in maintaining cell wall homeostasis.

## Discussion

Many bacteria remodel and recycle as much as 30 to 50% of the PG cell wall polymer during one generation of growth (44). This process prevents the loss of a substantial carbon and nitrogen resource that would otherwise be lost, for use either as an energy source or for the synthesis of more PG. However, PG remodeling in mycobacteria remains unresolved. This is despite mycobacteria encoding the glycosidase, amidase, endopeptidase, and carboxypeptidase machinery to breakdown its PG, producing an array of fragments that have pivotal roles throughout the pathogen’s lifecycle (45). Specifically, the mycobacterial recycling route/s for the exogenous PG breakdown products are not well defined and whether these PG metabolites are reused, or not, is not clear. Although import systems to salvage GlcNAc in other bacterial systems are known, the fate of GlcNAc in mycobacteria is less clear and appears to be influenced by the conditions encountered (46, 47). One potential route for mycobacteria to recover its PG is *via* the highly conserved *Mtb* UspABC ATP-binding cassette (ABC)-transporter, which has been implicated in the recognition of amino sugars (48). Yet little is known regarding the identity of other mycobacterial PG recycling systems as there are no obvious orthologues encoded within the mycobacterial genome. Furthermore, computational analyses suggest that PG salvage pathways differ between mycobacterial species, making it more difficult to infer function. For instance, the organization of the NagA PG recovery pathway varies among different species (Fig. S1). In *M. smegmatis* a putative amino PTS, PtsG-Crr, system is present within the genetic locus encoding *nagA*, pointing toward an additional route for GlcNAc reuse whereas no obvious PTS exist in *Mtb* (14, 49). In contrast, *ptsG* and *crr* are absent in *Mtb*, and instead *sugI* is located adjacent to *nagA*. SugI is a putative major facilitator superfamily transporter, so may have a role in amino sugar assimilation in *Mtb*, and further experiments are underway to investigate this further. Whether additional import systems for GlcNAc and/or other PG fragments exist remains to be elucidated.

To investigate PG recycling in mycobacteria we characterized the role of the highly conserved NagA enzyme as part of an unexplored, potential pathway for PG recovery (Fig. 1). In this study, a combination of genetic and biochemical studies revealed that NagA does indeed have a key role in controlling the fate and recovery of exogenous GlcNAc in *M. smegmatis* and is required for normal cell wall synthesis and biofilm formation. Even though NagA has a key role that acts as a gatekeeper between the cell wall biosynthesis and glycolysis pathways, it is solely responsible for catalyzing the deacetylation of GlcNAc-6-P to the essential GlcN-6-P amino sugar in the cell and no other enzyme can substitute for this function. Furthermore, our combined growth, uptake, and metabolite analyses all point toward an additional broader role of NagA (Figs. 2, 3 and 5). The inability of the *nagA* mutant to utilize GlcNAc as a sole carbon source (Fig. 2) was surprising given that NagA is not a transporter, which implies that NagA controls GlcNAc import *via* alternative mechanisms. A possible explanation for this emerged from our observation that the *nagA* mutant accumulates millimolar levels of GlcNAc-6-P in the absence of NagA (Fig. 5). As GlcNAc-6-P accumulation is toxic in *Escherichia coli* and *Streptomyces* (24, 50), it could therefore be envisaged that NagA tightly regulates the uptake and metabolism of GlcNAc as a self-protection mechanism to prevent the toxic build-up of GlcNAc-6-P in *M. smegmatis* cells. This process may potentially be driven by a self-amplifying feedback loop that signals to decrease GlcNAc uptake depending on the external and internal levels of GlcNAc and associated pathway metabolites. Our uptake experiments corroborate this observation as the rate and amount of ^14^C-GlcNAc uptake in the *nagA* mutant was significantly reduced (Fig. 3), whilst the slightly increased uptake of ^14^C-GlcNAc by the WT strain in the presence of GlcNAc may reflect induction of *nagA* under these conditions, which has been reported previously (33, 51). A similar phenotype has been observed for *Streptomyces* species where GlcNAc levels act as a master switch that signals for growth development and antibiotic production depending on the nutritional status of the environment (23, 52). This implies that both the environmental resources and the metabolic status of the intracellular amino sugar pool serve as a cue that regulates GlcNAc signaling and reuse in *M. smegmatis*. As GlcN-6-P can still be generated from the glycolysis pathway by the action of GlmS, it is intriguing that the intracellular levels of GlcN-6-P are not maintained (Fig. 5). Instead, the GlcN-6-P pool is completely depleted in the *nagA* deletion mutant (Fig. 5), below the limits of high-performance anion-exchange chromatography detection, highlighting that both the NagA and glycolysis pathways make important contributions in maintaining the intracellular reserves of GlcN-6-P. Because GlcN-6-P is an essential metabolite it is conceivable that mycobacteria cannot afford to retain surplus of GlcN-6-P when the NagA pathway is inactive. Instead, it is likely that once synthesized, GlcN-6-P is rapidly channeled into the cell wall, although it appears that GlcN-6-P levels are not sufficient to maintain proper cell wall homeostasis. GlcN-6-P levels are further reduced in Δ*nagA* by reprogramming of the glycolysis pathway (Fig. 7). GlmS is downregulated (FC: −1.4, *p*-value: 0.07), whereas the reverse reaction catalyzed by NagB is upregulated (FC: 1.8, *p*-value: 0.02) (Fig. 7) thereby reducing the flux of GlcN-6-P into cell wall biosynthesis. Hence, the crosstalk between cell wall biosynthesis and glycolysis, controlled by NagA, leads to diminished GlcN-6-P availability, which may cause an imbalance in GlcN-6-P consumption in these interlinked and competing pathways. An important consequence of impeded NagA activity is the altered fate of the GlcNAc metabolite. In the *nagA* mutant the ^14^C-GlcNAc that is imported accumulates at very high levels in the cytosol rather than in the cell wall (Fig. 3). The pronounced difference in the ^14^C-label incorporation pattern in Δ*nagA* was not as expected, with comparable ^14^C-levels within the PG and the AG linker unit (Fig. 3), implying significant defects in cell wall biosynthesis. Given that AG anchoring to PG is essential for mycobacterial survival (53) our findings imply that when the NagA PG recycling pathway is disrupted, mycobacteria prioritize synthesis of the entire AG linker unit over PG to prevent disruption of this extremely vulnerable pathway. This correlates with Δ*nagA’s* increased sensitivity to antimicrobials (Table S1), particularly PG targeting agents. Indeed, our PG fragment analysis and TEM studies further support this, revealing substantial structural and compositional changes in the mutant’s PG and a reduction in cell size (Fig. 4). We suggest that the compromised cell wall integrity in the *nagA* mutant triggers a compensatory mechanism that reinforces the PG layer, resulting in its thickening and increased peptide cross-linking to maintain cell fitness. In addition, beyond the PG layer, the *nagA* mutant exhibits a much thinner mycolyl-arabinogalactan layer (Fig. 4). The reduction is likely due to alterations in the PG structure, which may limit the availability of AG attachment sites, leading to decreased incorporation of AG in the cell wall. Together with the *nagA* mutant’s increased sensitivity to ethambutol (Table S1), a front-line TB drug that targets AG biosynthesis, these findings indicate that NagA also has a role in modulating the composition of mycobacterial cell wall AG.

In addition, the *nagA* deletion strain exhibited a severe defect in biofilm formation (Fig. 6). Although the extracellular polymeric substance composition of mycobacterial biofilms is not yet well-defined, sugar content analyses have identified the presence of mycobacterial cell wall sugars, including the released GlcNAc PG fragment, as integral components of this extracellular matrix (54). Our data indicate that high GlcNAc levels impair the formation of this structure, eliciting fragile biofilms with an altered architecture in WT *M. smegmatis,* highlighting the importance of nutrient and stress conditions on biofilm structure and composition. As we have established that NagA orchestrates GlcNAc recovery and reuse, we speculate that the formation of disrupted biofilms is linked to an accumulation of GlcNAc within the extracellular matrix combined with Δ*nagA*’s inability to recycle PG. Since disruption of other enzymes in the mycobacterial PG synthesis pathway also display defective biofilms (38, 39, 55), more work is now needed to unravel the exact role and molecular mechanisms of PG recycling in biofilm establishment and formation.

In conclusion, we have revealed that NagA has a pivotal role in salvaging PG in mycobacteria, providing a specialized pathway for these bacteria to recover scarce energy resources within a nutrient restricted environment. Loss of GlcNAc-6-P deacetylase activity creates a major bottleneck in the recovery of remodeled PG fragments, which leads to reprogramming of amino sugar flux and impedes a myriad of cellular processes. Given NagA’s crucial role in cell wall and biofilm formation, the development of NagA inhibitors combined with molecules that exploit the defective cell envelope or exhibit increased efficacy in the presence of defective biofilms could enhance the effectiveness of TB therapy, opening new avenues to explore to combat this major global pathogen.

## Experimental procedures

### Bacterial strains and culture conditions

*M. smegmatis* mc^2^155 strains were routinely cultured aerobically in either Luria-Bertani broth supplemented with 0.05% (vol/vol) Tween 80, Middlebrook 7H9 broth (Difco) supplemented with 0.2% (vol/vol) glycerol and 0.05% (vol/vol) Tween 80 (7H9), Sauton minimal media (0.5 g/L K_2_HPO_4_, 0.5 g/L MgSO_4_, 4.0 g/L asparagine, 2.0 g/L citric acid, 0.05 g/L ferric ammonium citrate, 0.0001% (wt/vol) ZnSO_4_ and 0.05% (vol/vol) tyloxapol containing the carbon source of interest supplemented with a defined carbon source at the indicated concentrations or Sauton-glycerol media (Sauton minimal media containing 5% (vol/vol) glycerol). Strains were routinely maintained on LB agar or Middlebrook 7H10 agar (Difco) supplemented with 10% (vol/vol) oleic acid-albumin-dextrose-catalase and 0.2% glycerol at 37 °C. Hygromycin (50 μg/ml) and kanamycin (25 μg/ml) were used when required. For growth on defined carbon sources, strains were cultured to mid-log phase and starved for 24 h in PBS supplemented with 0.05% tyloxapol (PBST) before inoculation into Sauton minimal media supplemented with the appropriate carbon source. For cloning procedures *E. coli* Top10 cells were grown in LB or on LBA supplemented with hygromycin (150 μg/ml) and kanamycin (50 μg/ml).

### Generation of the *nagA* gene-deletion mutant

The *nagA* deletion mutant was achieved using the phage based specialized transduction method. The allelic exchanges substrate for *nagA* was generated in the digested p0004s vector (a gift from Professor William R. Jacobs Jr, Albert Einstein College of Medicine, USA). Two DNA fragments corresponding to ∼ 1000 bases upstream and downstream of *nagA* were PCR amplified from *M. smegmatis* genomic DNA using the primers (MSMEG2119_LL, MSMEG2119_LR, MSMEG2119_RL, and MSMEG2119_RR) listed in Table S4. The PCR products were digested with *Alw*NI and ligated with the *hyg*^*R*^-*sac*B cassette and *oriE-cos* fragments released from the *Van*91I-digested p0004S vector. The allelic exchange plasmid: *nagA*_p0004s, was verified by DNA sequencing, using the primer pairs HL/OL and HR/OR (Table S4). The resulting knockout plasmid was linearized with *Pac*I and cloned into phasmid phAE159, as described (56). Allelic exchange in *M. smegmatis* was achieved by specialized transduction using hygromycin for selection, resulting in the replacement of *nagA* with the *γδres-sacB-hyg-γδres* cassette. The *M. smegmatis* Δ*nagA* mutant strain was confirmed by whole-genome sequencing of isolated genomic DNA (MicrobesNG). The sequencing data generated in this study have been deposited in the European Nucleotide Archive (ENA) under the accession number PRJEB90657. The data are available at https://www.ebi.ac.uk/ena/browser/view/PRJEB90657.

### Growth point monitoring of *M. smegmatis*

The growth of *M. smegmatis* strains in indicated growth media at 37 °C was monitored by measuring the absorbance at 600 nm (*A*_600_) at the time points indicated. The *M. smegmatis* starting *A*_600_ was 0.05. All experiments were undertaken in triplicate. Data were analyzed in GraphPad Prism (v10.1.1), and statistical significance was determined by Holm-Šídák multiple unpaired *t-*tests for each experimental group comparing Δ*nagA* to the WT control.

### NagA activity assay

*M. smegmatis* cultures were grown in 7H9 broth to an *A*_600_ = ∼1, washed twice in 0.1 M TES, pH 7.5, sonicated (amplitude = 8, 30 s on, 30 s off, 4 °C (Soniprep 150 Plus Ultrasonic Disintegrator)) and the clarified lysate obtained by centrifugation (16,000*g*, 10 min, 4 °C). Deacetylase activity in cell lysates was determined as previously described (57). The assay (250 μl) contained clarified lysate (100 μl), 0.2 M sodium phosphate buffer pH 7.5 (50 μl) and final concentrations of 10 mM *N*-acetylglucosamine-6-phosphate, 2 mM NADP, 4 U phosphoglucose isomerase, and 1.5 U glucose-6-phosphate dehydrogenase. The cell lysate assay was incubated at 37 °C for 60 min and the rate of formation of NADPH was determined spectrophotometrically at 340 nm (Tecan Infinite M200). Total lysate protein concentration was determined by a Bradford assay (590 nm, Tecan Infinite M200). Kinetic parameters were analyzed by nonlinear regression analysis (GraphPad Prism, v10.1.1) and expressed as mean ± standard deviation of triplicate measurements.

### Determination of ^14^C-GlcNAc uptake

*M. smegmatis* cells were grown to an *A*_600_ = 1 in Sauton minimal media (100 ml) supplemented with glucose (5 mM), or glucose (5 mM) and GlcNAc (1 mM). The cells were harvested by centrifugation (3220*g*, 10 min, 4 °C), washed three times in PBST and resuspended in PBST (300 μl) to give an *A*_600_ of ∼266. Uptake assays were performed at 37 °C with ^14^C-GlcNAc [glucosamine-14C(U)] (0.01 μCi/ml) (specific activity 250–360 mCi/mmol 9.25–13.32 GBq/mmol, American Radiolabeled Chemicals). Samples (30 μl) were taken at the indicated time points over 30 min. Uptake was terminated at 2.5 min, within the linear range, by the addition of 1 ml ice-cold PBST containing 500 mM GlcNAc (quenching buffer), followed immediately by centrifugation (16,000*g*, 10 min, 4 °C). The cell pellets were washed three times in ice-cold quenching buffer (1 ml) and then resuspended in 500 μl of the same buffer. The radioactivity corresponding ^14^C-GlcNAc uptake in the 500 μl sample was measured by scintillation counting in scintillation fluid (10 ml) (Ecoscint A, National Diagnostics). Each assay was performed in triplicate. ^14^C-GlcNAc uptake rates were determined at 2.5 min and expressed as mean ± standard deviation of triplicate measurements.

### ^14^C-GlcNAc labeling

Mid-log phase *M. smegmatis* strains (*A*_600_ = ∼0.5) were inoculated into Sauton minimal media supplemented with either glucose (5 mM) or glucose (5 mM) and GlcNAc (1 mM) to an absorbance of *A*_600_ = 0.1 before radiolabeling with 0.5 μCi/ml ^14^C-GlcNAc [glucosamine-14C(U)] (250–360 mCi/mmol 9.25–13.32 GBq/mmol, American Radiolabeled Chemicals) for 20 h at 37 °C. Cells were harvested by centrifugation (3220*g*, 10 min, 4 °C), washed five times with PBST supplemented with 500 mM GlcNAc and the cell fractions analyzed as described previously (33). Briefly, pellets were boiled in 4% SDS (500 μl) for 3 h, centrifuged (16,000*g*, 10 min, room temperature) and the supernatant retained as the cytosol sample. The pellet was subjected to boiling in 0.1 M HCl (500 μl) for 30 min to release the AG and GlcNAc containing linker fraction and the supernatant retained as the AG sample after centrifugation (16,000*g*, 10 min, room temperature). The remaining insoluble material in the pellet represents the peptidoglycan and was resuspended in water (500 μl). Samples (500 μl) were measured for radioactivity by scintillation in scintillation fluid (10 ml) (Ecoscint A, national diagnostics) All assays were performed in triplicate. Data were analyzed in GraphPad Prism (v10.1.1) and statistical significance was determined by Holm-Šídák multiple unpaired *t-*tests for each experimental group comparing Δ*nagA* to the WT control.

### Determination of MICs

The MICs of all compounds were determined using the resazurin reduction microplate assay as described previously described (58). Briefly, *M. smegmatis* strains were grown to mid-log phase (*A*_600_ = 0.6) and approximately 5 × 10^5^ cells were incubated at 37 °C in 7H9 broth containing 2-fold serial dilutions of each compound in a 96-well flat-bottom microtiter plate. The plates were incubated without shaking for 24 h before addition of 25 μl resazurin (one tablet of resazurin (VWR) dissolved in 30 ml of sterile PBS supplemented with 10% (vol/vol) Tween-80). Following a further 3 h incubation at 37 °C, the plates were assessed for color development. The MIC values were determined as the lowest concentration of drug that prevented the color change of resazurin (blue: no bacterial growth) to resorufin (pink: bacterial growth).

### Determination of drug susceptibility by spot assays

The percentage recovery of *M. smegmatis* WT and Δ*nagA* strains were determined against the compounds where two-fold differences in MIC values determined by resazurin reduction microplate assay were observed. WT and Δ*nagA* strains were grown to mid-log phase (*A*_600_ = 0.6). The cells were serially diluted in 7H9 media and spotted (10 μl) at 10^6^ to 10^1^ cells/well in 24-well plates containing 7H10 media (1 ml) with or without compound at concentration dilutions around the determined MIC values. The plates were incubated at 37 °C for 48 h, the colony forming units counted and CFU/ml calculated. The relative percentage recovery is expressed as a fraction compared to the CFU/ml of the untreated strains. All assays were performed in triplicate.

### Lysozyme susceptibility assay

*M. smegmatis* strains were grown to early log phase (*A*_600_ = 0.25) in 7H9 media. In addition, 100 μl of this culture was added to a 96 well-plate with the addition of 200 μg/ml lysozyme (final concentration) and incubated at 37 °C for 3 h, at which point samples were taken for CFU enumeration. The percentage survival was calculated by comparison to CFUs from a no lysozyme control. All assays were carried out in triplicate. Data were analyzed in GraphPad Prism (v10.1.1), and statistical significance was determined by a two-tailed *t* test.

### Ethidium bromide uptake

Mid-log phase *M. smegmatis* cultures (*A*_600_ = 0.5) grown in 7H9 broth were harvested by centrifugation (3220×*g*, 10 min, 4 °C), washed in PBST and resuspended in PBST to an *A*_600_ = 0.8. Uptake assays were performed with 100 μl cell suspension and a final concentration of 2 μg/ml ethidium bromide and the fluorescence monitored at λ_ex_ 535 nm, λ_em_ 595 nm (Tecan Infinite F200). Samples were taken every 10 min for a period of 60 min. The change in fluorescence was calculated by subtracting the fluorescence at time = 0 from the end point time = 60 min. Ethidium bromide uptake assays were carried out in triplicate. Data were analyzed in the Superviolin package in Python 3 (Minicondia), and statistical significance was determined by a two-tailed *t* test.

### Congo red binding

*M. smegmatis* strains (5 ml) were grown in triplicate for 3 days at 37 °C in 7H9 broth and 100 μg/ml congo red before harvesting (3220*g*, 10 min, 4 °C). The cells were then washed at least 5 times with water (10 ml) until the supernatant became clear. The pelleted cells were resuspended in water (1 ml), the *A*_600_ determined, and the cells then pelleted by centrifugation (3220*g*, 10 min, 4 °C). The cells were then incubated with dimethyl sulfoxide (1 ml) for 4 h at room temperature, with shaking. The cells were then pelleted (3220*g*, 10 min, 4 °C), and the absorbance of the dimethyl sulfoxide extract measure at 488 nm (A_488_). The Congo red binding index was calculated as a measure of A_488_ divided by the *A*_600_. All assays were performed in triplicate. Data were analyzed in GraphPad Prism (v10.1.1), and statistical significance was determined by a two-tailed *t* test.

### Growth conditions for peptidoglycan extraction, flow cytometry analysis, and transmission electron microscopy

*M. smegmatis* strains were grown to exponential phase (*A*_600_ ≈ 1.2) in LB media containing 0.05% Tween 80 (100 ml). The cells were harvested by centrifugation (3220*g*, 10 min, 4 °C), washed three times in PBST, and the pelleted cells resuspended in PBST (100 ml). The cells were then starved for 24 h at 37 °C with shaking, centrifuged (3220*g*, 10 min, 4 °C), resuspended in Sauton minimal media and inoculated into Sauton minimal media supplemented with either glucose (5 mM) or glucose (5 mM) and GlcNAc (1 mM) to an absorbance of *A*_600_ = 0.05. The cells were grown to either mid-log (*A*_600_ = ∼0.6) or stationary phase (48 h, *A*_600_ = ∼1.3), centrifuged (3220*g*, 10 min, 4 °C) and washed three times in PBST and the cell pellet retained for either PG extraction, imaging flow cytometry analysis or TEM.

### Peptidoglycan extraction

Cell pellets from 3 × 1L were resuspended in PBS (10 ml per 1L pellet) and lysed by sonication on ice (10 × 30 s on, 30 s off, Sonicator Ultrasonic Liquid Processor XL; Misonix). The lysates were combined and then subjected to boiling in 4% SDS to release the mAGP complex, and the insoluble mAGP collected by centrifugation (20,000*g*, 30 min, room temperature) and washed 10 times with water (35 ml) to remove the SDS (20,000*g*, 20 min, room temperature). The isolated mAGP was then resuspended in 0.5% (w/v) KOH in methanol and incubated at 37 °C for 96 h at 180 rpm to cleave the mycolic acids. The insoluble material containing the cleaved mycolic acids and the arabinogalactan-peptidoglycan complex was collected by centrifugation (3220*g*, 10 min, room temperature) and washed three times with methanol (15 ml) to remove the KOH. The cleaved mycolic acids were extracted from the mixture by washing three times with diethyl ether (15 ml, 3220*g*, 10 min, room temperature). The resulting arabinogalactan-peptidoglycan complex was then treated with 0.2 M H_2_SO_4_ at 85 °C for 30 min (without shaking) to release the AG. The mixture was then cooled and neutralized with NaHCO_3_. The insoluble PG was separated from the solubilized AG by centrifugation (3220*g*, 10 min, room temperature), washed with water three times, resuspended in 1 ml PBS (1 ml) and treated with DNase (20 mg/ml) and RNase (10 mg/ml) and incubated at 37 °C for 4 h at 600 rpm, before adding proteinase K (100 mg/ml) and incubated at 55 °C for 16 h, 600 rpm. SDS was then added to give a final concentration of 1% (wt/vol) and the samples boiled for 3 h before harvesting the PG by centrifugation (3220*g*, 10 min, room temperature) and washing the isolated PG with water 10 times (10 ml) to remove the SDS. The isolated PG samples were lyophilized and stored at −20 °C.

### Peptidoglycan digestion

One milligram of purified peptidoglycan was resuspended in 20 mM phosphate buffer, pH 5.5 (125 μl) supplemented with 200 U of mutanolysin (Sigma-Aldrich) and digested for 16 h, at 37 °C with agitation. Following heat inactivation of mutanolysin (5 min at 100 °C), soluble disaccharide peptides were mixed with an equal volume of 250 mM borate buffer (pH 9.25) and reduced with 0.2% (wt/vol) sodium borohydride for 20 min at room temperature, and the pH was then adjusted to 4.5 to 5.5 with phosphoric acid.

### LC-MS/MS data acquisition

An Ultimate 3000 UHPLC (Dionex) system coupled with a high-resolution Q Exactive Focus mass spectrometer (Thermo Fisher Scientific) was used for LC-MS/MS analysis. Muropeptides were separated using a C18 column (Hypersil Gold aQ, 1.9 μm particles, 150 mm × 2.1 mm; Thermo Fisher Scientific) at a temperature of 50 °C. Muropeptide elution was performed at 0.25 ml/min with mixture of solvent A (water, 0.1% [vol/vol] formic acid) and solvent B (acetonitrile, 0.1% [vol/vol] formic acid). LC conditions were 0 to 12.5% B for 25 min increasing to 20% B for 10 min. After 5 min at 95%, the column was re-equilibrated for 10 min with 100% buffer A. The Orbitrap Exploris 240 was operated under electrospray ionization (H-ESI high flow)-positive mode, full scan (m/z 150–2250) at resolution 120,000 (FWHM) at *m/z* 200, with normalized AGC Target 100%, and automated maximum ion injection time (IT). Data-dependent MS/MS were acquired on a ‘Top 5’ data-dependent mode using the following parameters: resolution 30,000; AGC 100%, automated IT, with normalized collision energy 25%.

### LC-MS/MS data analysis strategy

A preliminary search was carried out to identify disaccharide-peptides present across all datasets using the proprietary software Byos. We searched LC-MS/MS datasets using a database containing 156 muropeptides (DB1; Supplementary File S1) including di, tri, tetra and pentapeptide stems containing A, E, Q, *m*-DAP (amidated or not) and noncanonical residues at their C terminus. These peptide stems were searched with N-terminal modifications corresponding to various disaccharide-peptides including GlcNAc-MurNAc (gm) as well as their glycolylated (gm(Glyc) and deacetylated variants gm(DeAc). The automated MS/MS analysis did not identify any noncanonical residues. It confirmed the presence of glycolylated MurNAc and deacetylated sugar residues, as well as the presence of peptide stems containing Glu or Gln and both m-DAP (referred to as J) and amidated m-DAP (referred to as Z). Using this information, we next built a database containing 24 monomers (DB2; Supplementary File S1) containing the gm(Glyc) and gm disaccharides moieties linked to peptide stems containing A,E,Q,J and Z (AEJAA, AQJAA, AEZAA, and AQZAA). DB2 was used to perform searches using the open-source software PGFinder (59, 60). Datasets were deconvoluted using Byos, and searches were carried out using a 5 ppm tolerance, allowing the search for deacetylated variants. A total of 29 monomers (including eight deacetylated ones) were identified across all samples (Supplementary File S2). The search output was chosen to identify the eight most abundant monomers that could be used as a donor or an acceptor (gm-AEJ, gm-AEJA, gm(Glyc)-AEZ, gm(Glyc)-AEZA, gm(Glyc)-AEJ, gm(Glyc)-AEJA, gm(Glyc)-AQZ, and gm(Glyc)-AQZA). These eight muropeptides were used to build 64 dimers. A total of 20 dimers were identified across all samples. All monomers from DB2, the eight deacetylated monomers identified by PGFinder and the 64 dimers built were combined to generate a final database called DB3 (Supplementary File S1). DB3 was used for a “one off” search (Supplementary File S3).

### Determination of cell size by flow cytometry

Cell pellets were washed once with PBST and resuspended in PBST to an *A*_600_ of 0.2. Samples were imaged using multispectral imaging flow cytometry (CYTEK ImageStream^X^ MkII, Amnis Corporation) acquiring 30,000 events per sample across three biological repeats. Imaging was performed using the 60× magnification lens, with the 488 nm laser (100 mW) for brightfield channel imaging. Data were analyzed using IDEAS software (IDEAS v6.3, Amnis Corporation). Single rod-shaped cells were gated based on the “area” *versus* aspect ratio plot of the brightfield image (aspect ratio: cell minor axis divided by cell major axis). After gating ∼10,000 single rod-shaped WT cells and ∼10,000 Δ*nagA* cells were measured. Cells were masked using the “Adaptive Erode” mask with a 74-pixel threshold. This cutoff was selected as the highest threshold that masked the cell (Fig. S11). Cell length was derived from this mask using the “length” feature. Data were analyzed in GraphPad Prism (v10.1.1), and statistical significance determined by an unpaired *t* test.

### Analysis of cells by TEM

Cell pellets were resuspended in PBS containing 2.5% glutaraldehyde and incubated at room temperature for 1 h, centrifuged (10 min, 3220*g*, 4 °C), washed once with PBST and washed three times in water. The cells were resuspended in 1% (wt/vol) osmium tetroxide for 1 h to stain and then washed. After stepwise dehydration in 25%, 50%, 75%, and 100% (vol/vol) acetone, the cells were infiltrated with 50% (wt/vol) resin (Agar LV resin) for 1 h followed by 100% resin for 24 h. The Agar LV resin was cured at 60 °C overnight. After ultrathin sectioning on an RMC ultramicrotome, sections were post stained in 2% (wt/vol) uranyl acetate and 1.5% (wt/vol) lead citrate. The samples were imaged in a JEOL JEM2100Plus with Gatan OneView CMOS camera. Images were processed in ImageJ using the “plot profile” feature.

### Metabolite analysis by ion chromatography

Strains were cultured in Sauton-glycerol media (200 ml) to *A*_600_ = ∼1.0 with or without the addition of GlcNAc (20 mM). Cells were harvested by centrifugation (3220*g*, 10 min, 4 °C), washed with PBST twice, snap frozen, resuspended in water, lysed by sonication (amplitude = 8, 30 s on, 30 s off, 4 °C, Soniprep 150 Plus Ultrasonic Disintegrator) and centrifuged (16,000*g*, 10 min, 4 °C). The supernatant was lyophilized before resuspension in 500 μl 18 MΩ H_2_O, filtered through a 10-kDa molecular weight cutoff centrifuge filter (Amicon) and the filtrate analyzed. Samples were analyzed by high-performance anion-exchange chromatography on a Dionex ICS5000+ system with a CarboPac PA-20 analytical column (3 mm × 150 mm) and PA-20 guard column (3 mm × 30 mm) kept at 30 °C. Detection was by pulsed amperometry with standard quadrupole waveform. Multistep gradient elution was performed as shown in Table 2 (eluent A = 18.2 MΩ H_2_O, eluent B = 100 mM NaOH, eluent C = 100 mM NaOH, 800 mM NaOAc) with a total run time of 50 min. Authentic standards of *N*-acetylglucosamine, *N*-acetylglucosamine-6-phosphate, glucosamine-6-phosphate were run for comparison. Chromeleon 7 software (Dionex) was used for data processing. To quantify uptake, the peak area of the GlcNAc, GlcNAc-6-P, and GlcN-6-P standards at varying concentrations (GlcN: 0–10 μM, GlcNAc: 0–25 μM, GlcN-6-P: 0–100 μM, GlcNAc-6-P 0–100 μM) were measured (Chromeleon 7 software). The peak area was plotted against concentration and simple linear regression plotted. To determine the concentration of these metabolites in cytosolic samples, the area of the peaks of interest was measured (Chromeleon 7 software) and the concentration determined from the calibration plot. Data were analyzed in GraphPad Prism (v10.1.1), and the statistical significance was determined by Holm-Šídák multiple unpaired *t-*tests for each experimental group comparing Δ*nagA* to the WT control.

**Table 2:** High-performance anion-exchange chromatography KOH elution gradient

Time (mins)	% Eluent A (H_2_O)	% Eluent B (100 mM NaOH)	% Eluent C: (100 mM NaOH-800 mM NaOAc)	Flow rate (mL/min)
0	95	5	0	0.5
5	95	5	0	0.5
20	50	50	0	0.5
35	50	25	25	0.5
40	50	25	25	0.5
41	50	50	0	0.5
43	95	5	0	0.5
50	95	5	0	0.5

### Biofilm generation and quantification

*M. smegmatis* strains were cultured in Sauton-glycerol media supplemented with 0.05% Tween-80 at 37 °C, with shaking to mid-log phase (*A*_600_ = 0.6). Cultures were diluted to *A*_600_ = 0.03 in detergent free Sauton-glycerol medium with or without 20 mM GlcNAc and seeded into 24 or 96 well plates. Pellicles were incubated without shaking at 30 °C for 7 days before imaging, biofilm quantification and CFU enumeration. Crystal violet assays were performed to quantify biofilm biomass. Media were removed from the well, and the remaining biofilm was dried at 37 °C and stained with crystal violet (1 ml 0.1% (wt/vol) crystal violet solution). After 15 min incubation at room temperature the wells were washed with water (3 × 1 ml) and crystal violet extracted in ethanol (1 ml) followed by measurement of the absorbance of the solution at 600 nm (Tecan Infinite M200). CFUs were determined from biofilms cultured in 96-well plates by the addition of Tween-80 to a final concentration of 0.1% to each well and incubation for 30 min at room temperature before homogenizing the biofilm by pipetting. Wells were incubated for a further 10 min at room temperature before repeating homogenizing by pipetting. The solution was 10-fold serially diluted, the dilutions plated onto LBA, incubated at 37 °C for 3 days and CFUs determined. All assays were performed in triplicate. Data were analyzed in GraphPad Prism (v10.1.1) and statistical significance was determined by Holm-Šídák multiple unpaired *t-*tests for each experimental group comparing Δ*nagA* to the WT control.

### Proteomic sample preparation and analysis

*M. smegmatis* strains (WT and Δ*nagA,* 30 ml) were grown to an *A*_600_ of 0.8 in 7H9 media. The cells were harvested (3550*g*, 20 min, 4 °C), washed (3 × PBST) and the pellet resuspended in lysis buffer (PBS, 1 mM DTT, 1 mg/ml lysozyme, protease inhibitor (Pierce) pH 7.4) for 2 h at room temperature. 0.1 mm silica glass beads were added, and the cells were disrupted by bead-beating (4 × 45 s on, 45 s off, placed on ice between cycles, 6 m/sec, FastPrep-24 5G (MP Biomedicals)) followed by sonication (water sonicator bath) at room temperature for 15 min. The samples were centrifuged (2300*g*, 20 min, 4 °C) and the supernatant collected. The protein concentration was determined by Qubit fluorometer (Invitrogen) using Qubit Protein Assay Kit (Invitrogen). Protein samples (15 μl) were mixed with 2 × SDS loading dye, loaded directly onto an SDS-gel (Bio-Rad Any kD Mini-PROTEAN TGX) and run for 5 min, and the excised gel bands prepared for proteomics analysis as described previously (61). In brief, samples were reduced with 10 mM tris-2(-carboxyethyl)-phosphine (TCEP), alkylated with 40 mM chloroacetamide (CAA) and then in-gel digested with trypsin (2.5 ng/ml), and the peptides extracted with 25% acetonitrile containing 5% formic acid. The extracted peptides were dried under vacuum to a volume of 20 μl and resuspended to a total volume of 50 μl in 2% acetonitrile, 0.1% trifluoroacetic acid. Mass spectrometry was performed on a Thermo Orbitrap Fusion (Thermo Fisher Scientific) coupled to an Ultimate 3000 RSLCnano HPLC (Dionex) using an Acclaim PepMap μ-precolumn cartridge (300 μm i.d. × 5 mm, 5 μm, 100 Å) and an analytical Acclaim PepMap RSLC column (75 μm i.d. × 50 cm, 2 μm, 100 Å, Thermo Fisher Scientific). Mobile phase buffer A was composed of 0.1% (vol/vol) formic acid in water, and mobile phase B was composed of acetonitrile containing 0.1% (vol/vol) formic acid. The gradient was programmed as follows: 4% B increased to 25% B over 90 min, then further increased to 35% B over 13 min, followed by 3 min 90% B with a flow rate of 250 nl/min. Survey scans of peptide precursors from 375 to 1575 *m*/*z* were performed at 120 K resolution (at 200 *m*/*z*) with a 2 × 10^5^ ion count target. The maximum IT was set to 150 ms. Tandem MS was performed by isolation at 1.2 Th using the quadrupole, higher-energy collisional dissociation fragmentation with normalized collision energy of 33, and rapid scan MS analysis in the ion trap. The MS^2^ ion count target was set to 3 × 10^3^ and maximum IT was 200 ms. Precursors with charge state 2 to 6 were selected and sampled for MS^2^. The dynamic exclusion duration was set to 45 s with a 10 ppm tolerance around the selected precursor and its isotopes. Monoisotopic precursor selection was turned on and instrument was run in top speed mode. The raw data were searched using MaxQuant with an integrated Andromeda search engine (V1.5.5.1) (62) against both the *M. smegmatis* database and the common contaminant database from MaxQuant. Peptides were generated from a tryptic digestion with up to two missed cleavages, carbamidomethylation of cysteines as fixed modifications, and oxidation of methionines as variable modifications. Precursor mass tolerance was 10 ppm and product ions were searched at 0.8 Da tolerances. For protein quantification, label-free quantification (LFQ) was selected and proteins with LFQ minimum ratio count of two were retained. The PSM false discovery rate (FDR), protein FDR and site decoy fraction were set to one for further analysis in Scaffold or to 0.01 for analysis in Perseus. Scaffold (version 4.6.2) was used to validate MS/MS based peptide and protein identifications. Peptide identifications were accepted if they could be established at greater than 95.0% probability by the Scaffold Local FDR algorithm. Protein identifications were accepted if they could be established at greater than 95.0% probability and contained at least two identified peptides. Proteins that contained similar peptides and could not be differentiated based on MS/MS analysis alone were grouped to satisfy the principles of parsimony. Proteins sharing significant peptide evidence were grouped into cluster. Data processing and annotation was performed used the Perseus module of MaxQuant version 1.6.2.2 (63). First, the reverse and contaminant hits (as defined in MaxQuant) were eliminated from the MaxQuant output files. Only protein groups identified with at least two uniquely assigned peptide and quantified with a minimum of two ratio counts were used for the analysis. For each experiment, the LFQ intensity was transformed using the binary logarithm (log_2_). Protein groups were considered reproducibly quantified if identified and quantified in at least two replicates, missing LFQ intensity scores were assigned from a normal distribution. Protein groups were assigned a probability value (*p*-value) using a two-sample Student’s *t* test. *p*-values were subject to a -log_10_ transformation. Proteins were considered significant if the *p*-value < 0.05 (-log_10_(*p*-value) greater than 1.30) and had a two-fold change in protein expression (log_2_(LFQ difference) greater than one or < −1). Protein function, product, functional category were assigned based on Mycobrowser (release 3) annotations (64). The mass spectrometry proteomics data have been deposited to the ProteomeXchange Consortium *via* the PRIDE partner repository with the dataset identifier PXD065120 and 10.6019/PXD065120.

## Data availability

All the data generated in this study can be shared upon request.

## Supporting information

This article contains supporting information.

## Conflict of interest

The authors declare that they have no conflicts of interest with the contents of this article.
