# Supplementary material for: The deacetylase NagA mediates the remodeling and recycling of peptidoglycan-derived amino sugars in mycobacteria
Source: J Biol Chem. 2025 Aug 14;301(11):110597. doi: 10.1016/j.jbc.2025.110597 (PMC12617635; doi:10.1016/j.jbc.2025.110597)
Supplement: Supporting Figures and Tables [file mmc1.pdf]

Supporting information

The deacetylase NagA mediates the remodelling and recycling of peptidoglycan-derived amino sugars in mycobacteria

Collette S. Guy[1], Charlotte Cooper[1†], Magdalena Karlikowska[1†], James Harrison[1], Albel Singh[2], Luis Steven Servín-González[1], Caroline A Evans[3], Saskia E. Bakker[1], Andrew Bottrill[1], Apoorva Bhatt[2], Stéphane Mesnage[4], Gurdyal S. Besra[2] and Elizabeth Fullam[1,5,6]*

[1] School of Life Sciences, University of Warwick, Coventry, CV4 7AL, UK
[2] Institute of Microbiology & Infection, School of Biosciences, University of Birmingham, Birmingham, B15 2TT, UK
[3]Department of Chemical and Biological Engineering, ChELSI Institute, University of Sheffield, Sheffield, UK
[4] School of Biosciences, University of Sheffield, Sheffield S10 2TN, UK
[5] Manchester Institute of Biotechnology, University of Manchester, Manchester M1 7DN, United Kingdom
[6] Department of Chemistry, University of Manchester, Manchester M13 9PL, United Kingdom.

*Email for correspondence: elizabeth.fullam@manchester.ac.uk Tel.+ +44 (0)161 3068204

[†]These authors contributed equally and listed in alphabetical order

Table of Contents

Supplementary Results
Fig S1. Genetic organisation of putative *nagA* containing operons in mycobacterial species 3
Fig S2. Construction and confirmation of the *M. smegmatis* Δ*nagA* mutant. 4
Fig S3. Monitoring of viability by CFU enumeration 5
Fig S4. Loss of NagA leads to lysozyme susceptibility 6
Fig S5. Two-dimensional lipid analysis of wild type and Δ*nagA* strains 7
Fig S6. Strategy for peptidoglycan structural analysis 8
Fig S7. TEM images of *M. smegmatis* wild type and ΔnagA cells grown to log phase in Sauton's minimal media + 5mM Glc. 9
Fig S8. TEM images of *M. smegmatis* wild type and ΔnagA cells grown to log phase in Sauton's minimal media + 5mM Glc + 1 mM GlcNAc. 10
Fig S9. TEM images of *M. smegmatis* wild type and ΔnagA cells grown to stationary phase in sauton's minimal media + 5mM Glc. 11
Fig S10. TEM images of *M. smegmatis* wild type and ΔnagA cells grown to stationary phase in sauton's minimal media + 5mM Glc + 1 mM GlcNAc. 12
Fig. S11. Flow cytometry imaging 13
Table S1. Minimum inhibitory concentrations (MIC) of compounds tested against WT *M. smegmatis* and Δ*nagA* 14
Table S2. Minimum inhibitory concentrations (MIC) of β-lactam compounds tested in the presence and absence of the β-lactamase inhibitor clavulanic acid (CLAV) against WT *M. smegmatis* and Δ*nagA*. 14
Table S3. Spot assay of compounds tested against WT *M. smegmatis* and Δ*nagA* 15
Table S4. Primers used in this study 16
Methods
RNA isolation and operon analysis 17
Lipid analysis 17
References 18

Fig S1. Genetic organisation of putative *nagA* containing operons in mycobacterial species. Genes are colour coded according to G+C content and grey colour indicates pseudogenes. *Adapted from* MycoDB Xbase

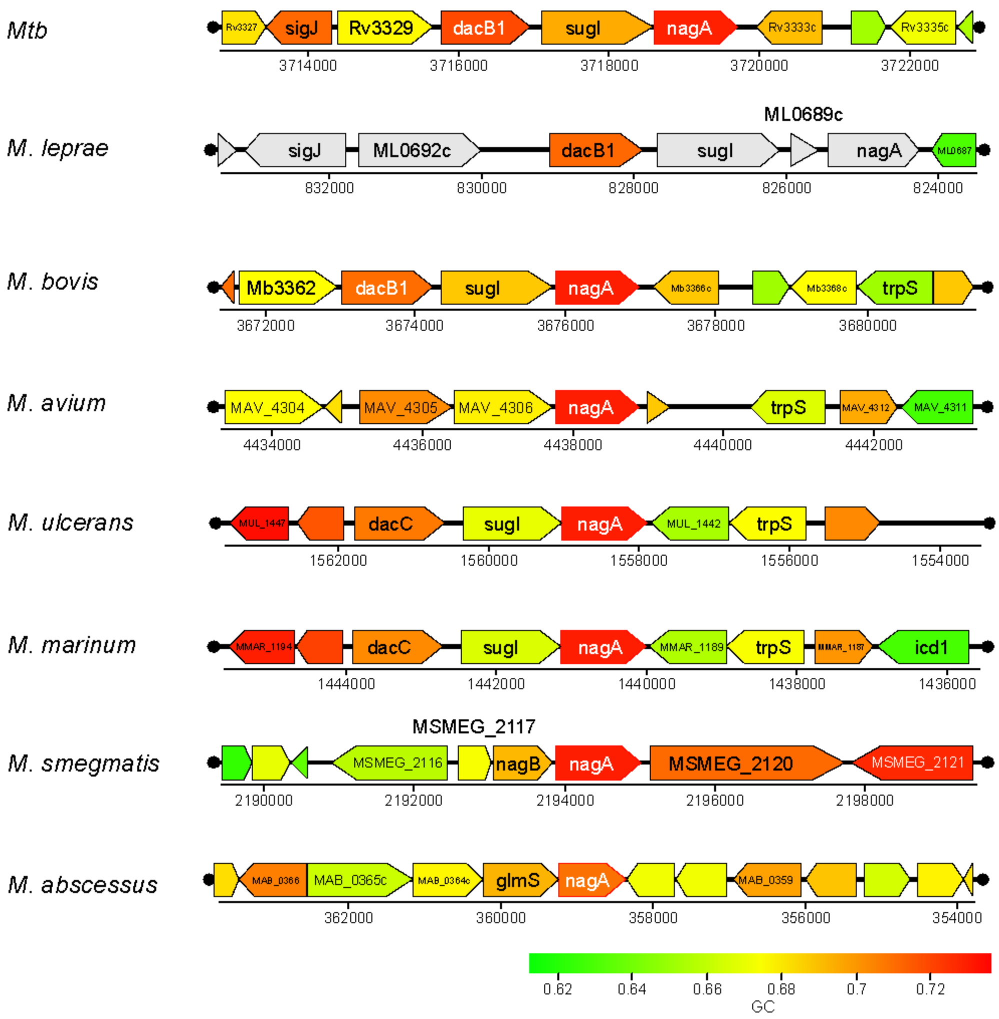

Fig S2. Construction and confirmation of the *M. smegmatis* Δ*nagA* mutant. **A)** Organisation of the *nagA* gene locus in *M. smegmatis* WT and the Δ*nagA* mutant. δres: res-sites of the δ-resolvase; hygR: hygromycin resistance gene. **B)** Whole genome sequencing analysis of Δ*nagA* confirming the deletion of the *nagA* gene **C-F)** RT-PCR analysis of genes within the *nagA* operon. **C)** *crr (*MS_2117) **D)** *nagB* **E)** *nagA* and **F)** the house-keeping gene *mysA*. Expression of *crr* and *nagB* genes within the *nagA* operon was confirmed in the wild-type and *nagA* deletion mutant. No expression of *nagA* was observed in the *nagA* deletion. 'g': genomic DNA, '-': RNA with no reverse transcriptase addition, '+' RNA with reverse transcriptase addition

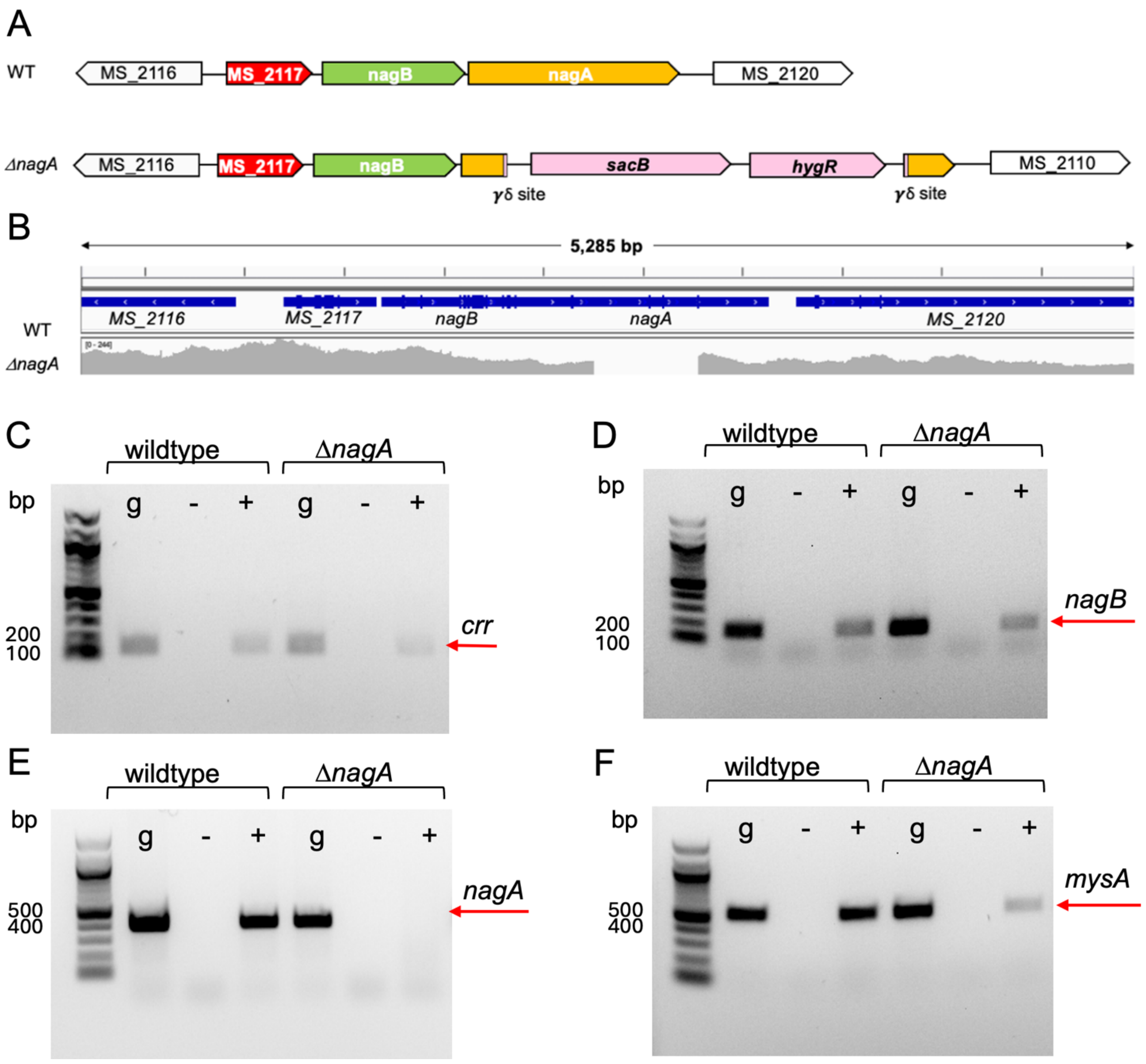

Fig S3. Monitoring of viability by CFU enumeration A) initial starvation in PBS supplemented with 0.05% tyloxapol and **B)** after incubation for 7 days in minimal media, or minimal media supplemented with GlcNAc (20 mM) as a carbon source. Error bars represent standard deviation from three biological replicates. Statistical significance was determined using unpaired *t*-tests, ns = not significant.

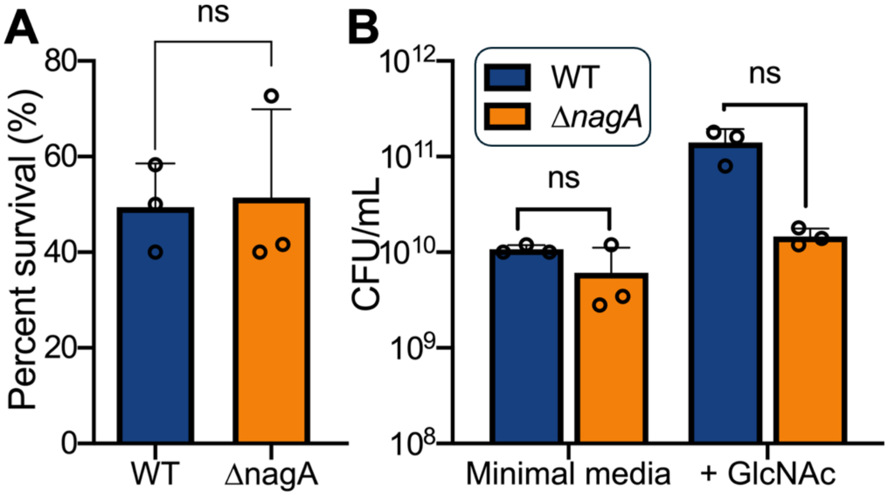

Fig. S4 Loss of NagA leads to lysozyme susceptibility but does not alter Ethidium bromide uptake or Congo red binding. A) Percentage survival of *M. smegmatis* WT and Δ*nagA* after exposure to lysozyme for 3 hr determined by CFU enumeration **B**) Ethidium bromide uptake of *M. smegmatis* WT and Δ*nagA* measured by a change in fluorescence (RFU) **C**) Congo red binding index. Error bars represent standard deviation from three biological replicates. Statistical significance was determined using a two-tailed t-test * = p<0.05, ns: not significant

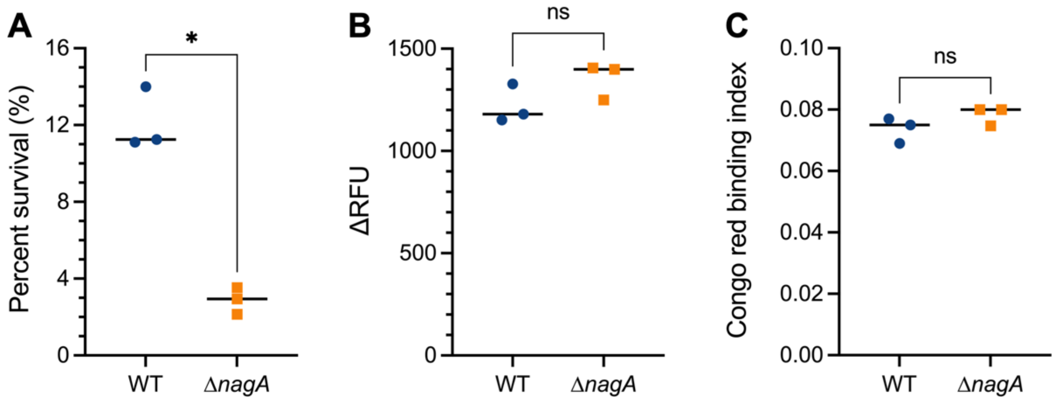

Fig S5. Two-dimensional lipid analysis of WT and Δ*nagA* strains. Apolar lipid extracts were loaded onto silica plates and run in **i)** System A: direction 1 (DI) separated 3x with petroleum ether 60-80/ethyl acetate (98:2) and direction 2 (DII) separated 1x with petroleum ether 60-80/acetone (98:2) **ii)** System B DI separated 3x with petroleum ether 60-80/acetone (98:2) and DII separated 1x with toluene/acetone (95:5) **iii)** System C DI separated 1x with chloroform/methanol (96:4) and DII separated 1x with toluene/acetone (80:20) **iv)** System D: direction 1; chloroform/methanol/water (100:14:0.8) 1x, direction 2; chloroform/methanol/water (60:30:6) 1x, direction 2; chloroform/acetone/methanol/water (50:60:2.5:3) 1x **iv)** System D DI separated 1x with chloroform/methanol/water (100:14:0.8) and DII separated 1x with chloroform/methanol/water (60:30:6). Fatty acid methyl esters (FAMEs) and mycolic acid methyl esters (MAMEs) were extracted and run in **v)** System F separated 3x with petroleum-ether 60-80/ acetone (19:1). The TLC plates were stained with 10% phosphomolybdic acid in ethanol**.** TAG = triacylglycerol, DAG = diacylglycerol, FA = fatty acid, MA = mycolic acid, GMM = glucose monomycolate, TMM = trehalose monomycolate, TDM = trehalose dimycolate, FAMEs = fatty acid methyl esters, MAMEs = mycolic acid methyl esters; α = alpha-MAME subclass, α = alpha-MAME subclass, e = epoxy-MAMEs.

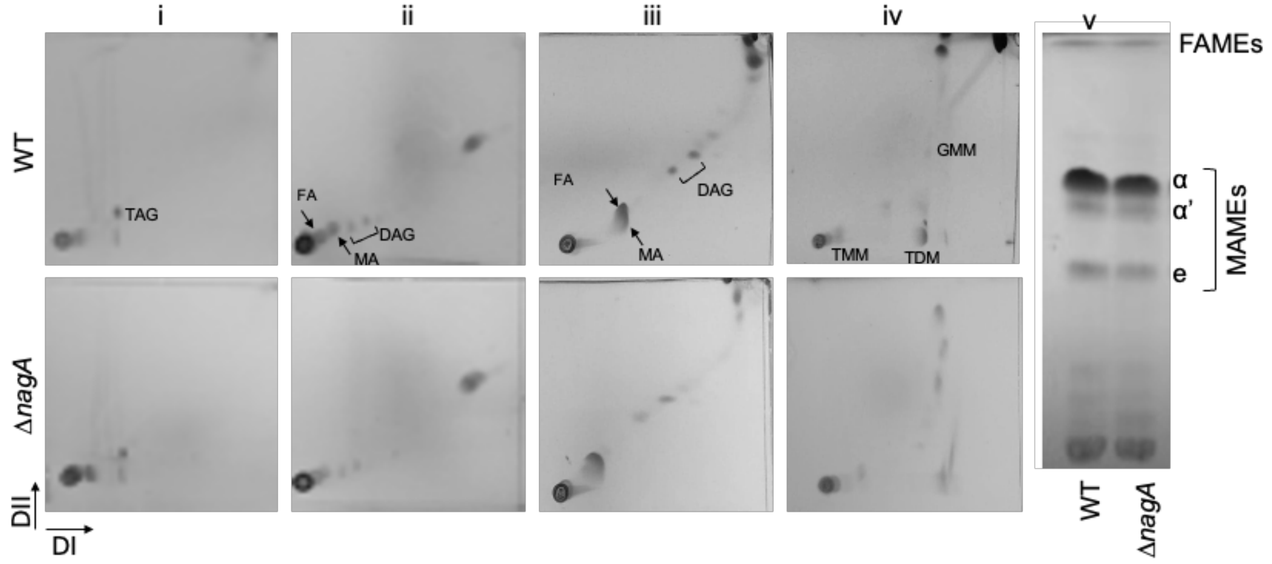

Figure S6. Strategy for peptidoglycan structural analysis. Sequential searches were performed using the Byonic™ and PGFinder software. The monomer database DB_1 was built based on the MS/MS analysis carried out with Byonic™. The identification of the most abundant monomers was used to build the databases containing 24 monomers (DB_2). A search with DB_2 identified 8 deacetylated monomers. DB_3 contained the 24 unmodified monomers from BD_2, the 8 deacetylated monomers identified and 64 dimers. These dimers corresponded to all possible combinations of the most abundant monomers that can be used as donors or acceptors. DB_3 was used for a final « one off » search.

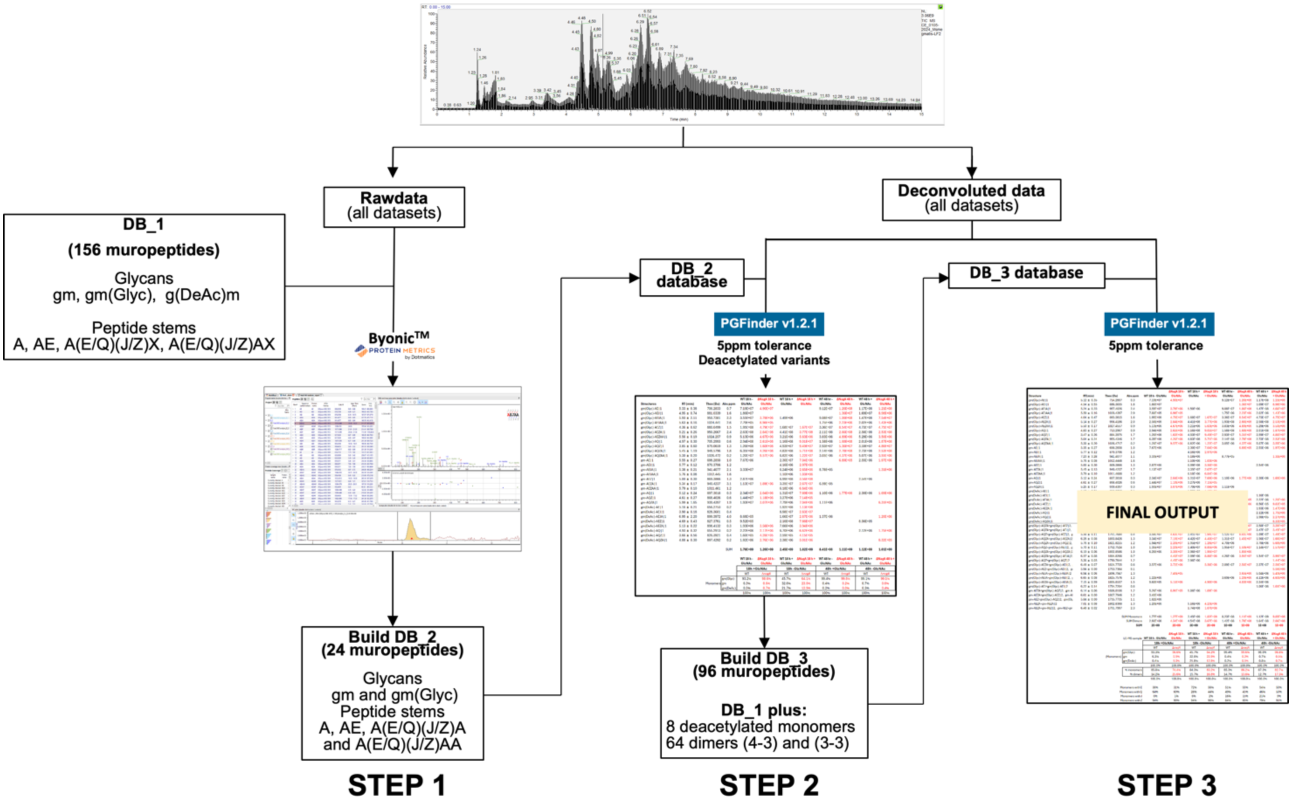

Fig. S7. Transmission electron microscopy (TEM) images of *M. smegmatis* WT and Δ*nagA* cells grown to log phase in Sauton's minimal media + 5mM Glc. **A**) WT, **B**) Δ*nagA*.

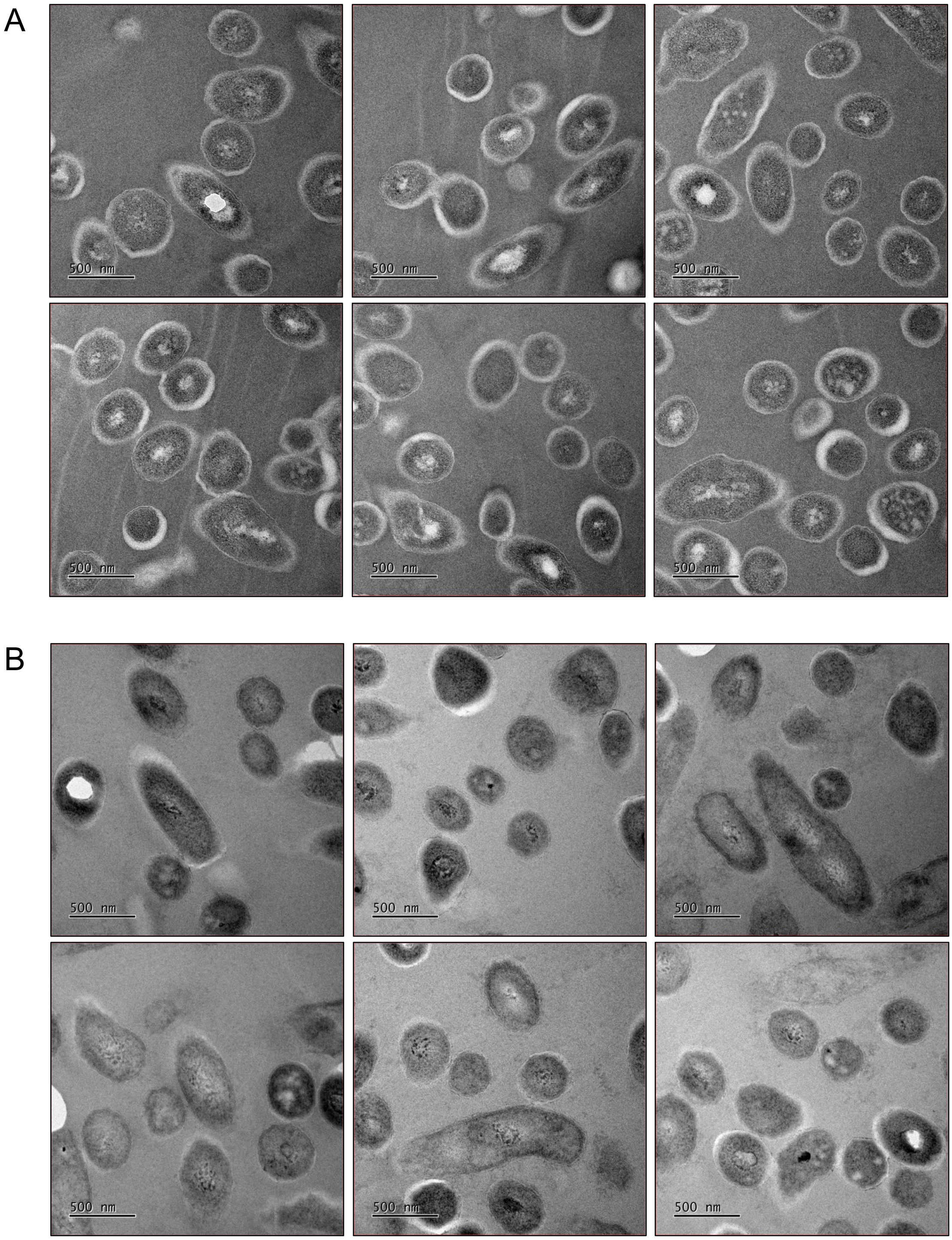

Fig. S8. Transmission electron microscopy (TEM) images of *M. smegmatis* WT and Δ*nagA* cells grown to log phase in Sauton's minimal media + 5mM Glc + 1 mM GlcNAc. A) WT, **B**) Δ*nagA*. Note that main paper Fig. 4B is shown here in A (second row, first panel) and that main paper Fig. 4C is shown here in B (top row, first panel). All TEM images are shown in full, without cropping. In panel B (second row), the first and second images partially overlap, with adjacent areas of the same section captured.

A

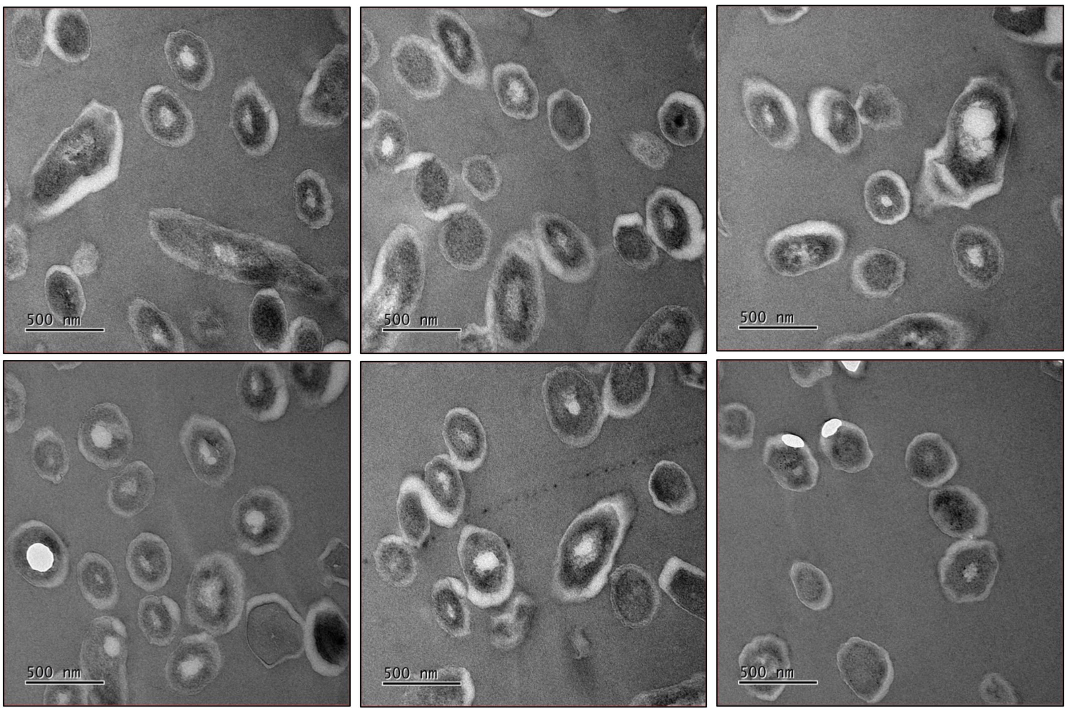

B

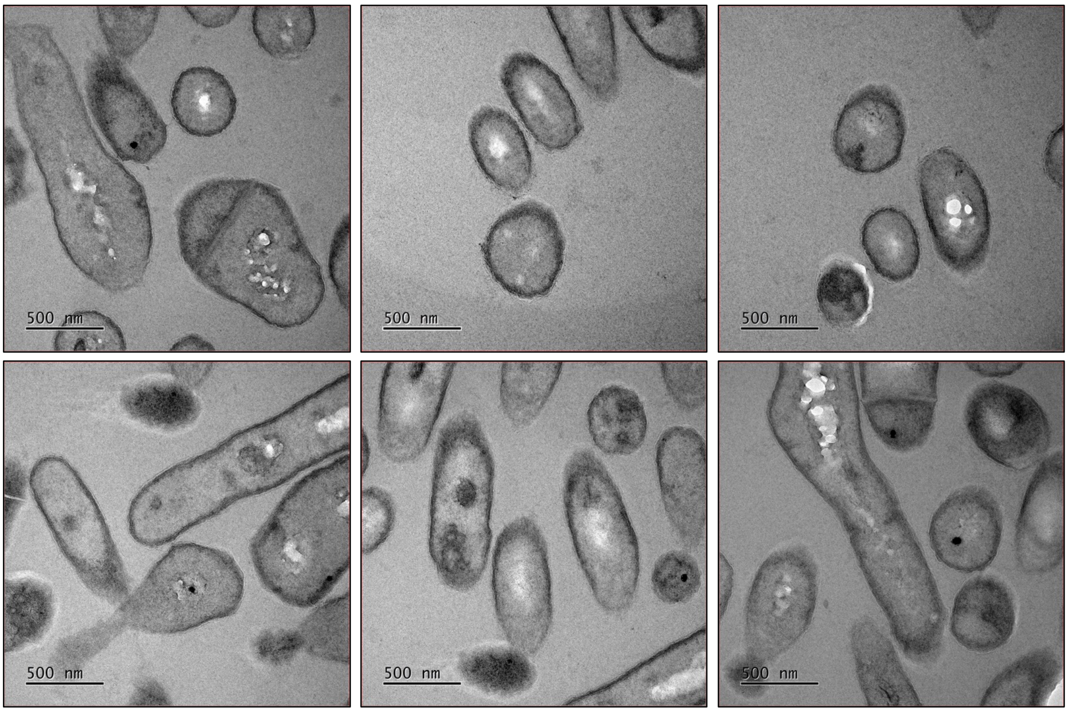

Fig. S9. Transmission electron microscopy (TEM) images of *M. smegmatis* WT and *ΔnagA* cells grown to stationary phase in Sauton's minimal media + 5mM Glc. **A**) WT **B**) Δ*nagA*. All TEM images are shown in full, without cropping. In panel B (second row), the first and third images partially overlap, with adjacent areas of the same section captured.

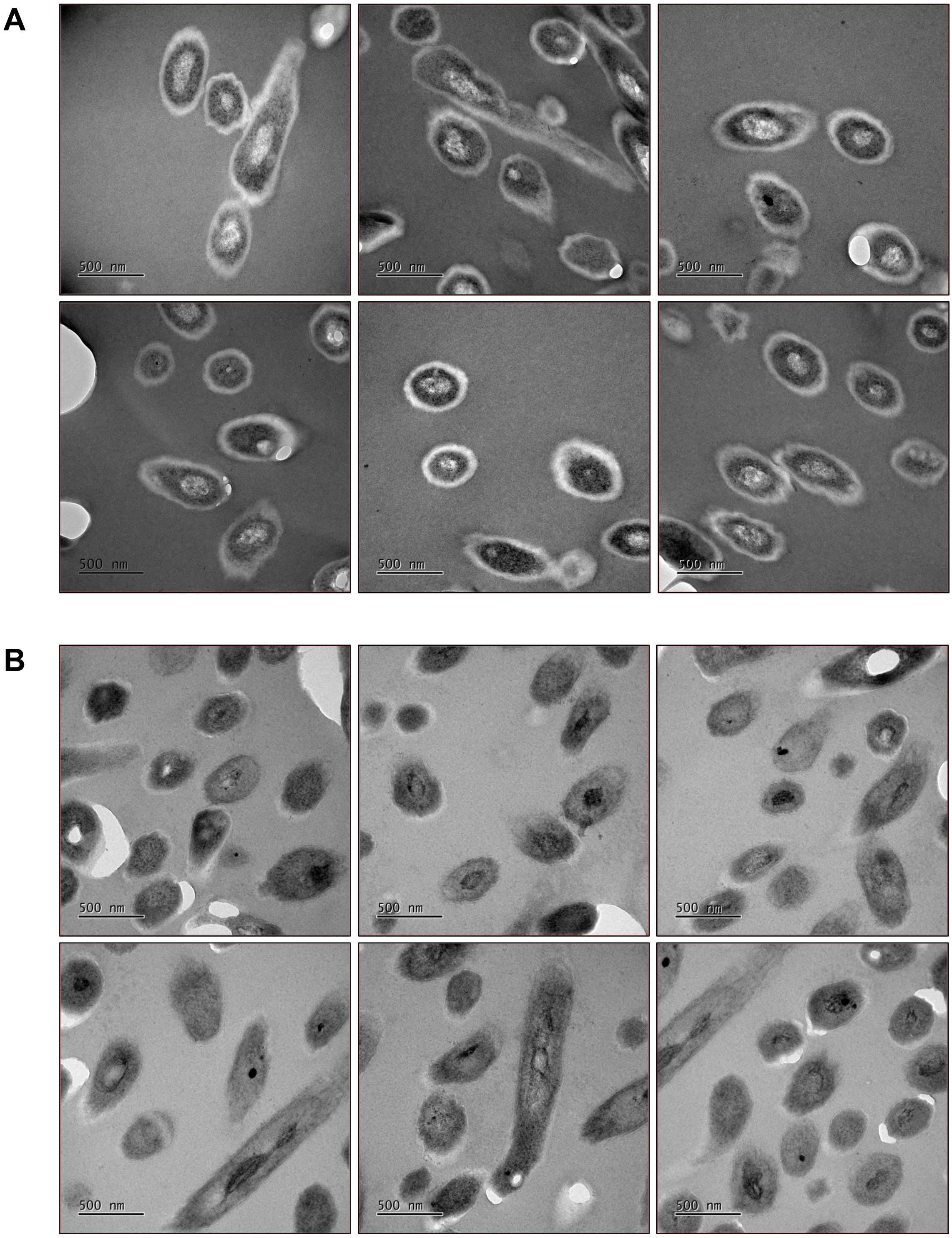

Fig. S10. Transmission electron microscopy (TEM) images of *M. smegmatis* WT and Δ*nagA* cells grown to stationary phase in Sauton's minimal media + 5mM Glc + 1 mM GlcNAc. **A**) WT, **B**) Δ*nagA*.

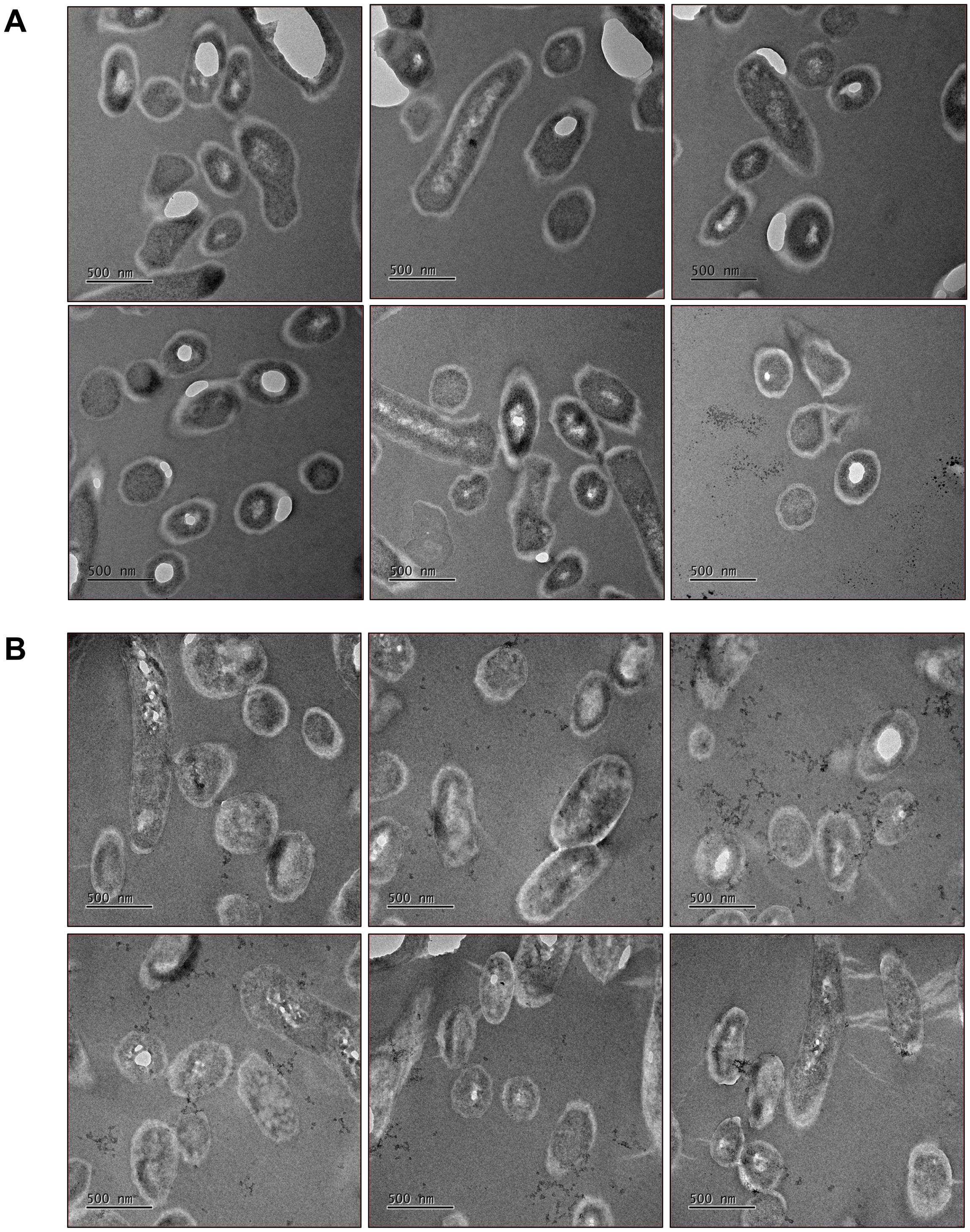

Fig. S11. Flow cytometry imaging. Representative flow cytometry images for **A)** WT, **B)** Δ*nagA* with the Adaptive Erode 74-pixel mask in blue.

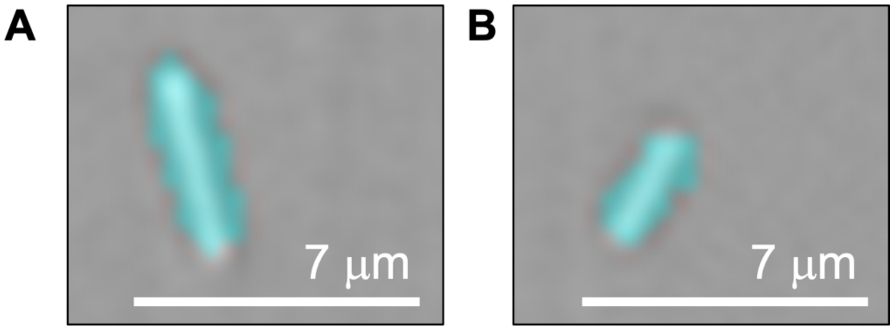

Table S1. Minimum inhibitory concentrations (MIC) of compounds tested against *M. smegmatis* WT and Δ*nagA*. MICs were determined as the lowest concentration of drug that prevented the colour change of resazurin (blue –no bacterial growth) to resorufin (pink –bacterial growth) and were performed in triplicate. Where values differed between replicates, a range is stated.

	**MIC (µg/mL)**	
**Compound**	**WT**	**Δ*nagA***
Apramycin	1.56 – 3.13	3.13
Gentamycin	0.78	0.39-0.78
Kanamycin	0.39 – 0.78	0.39 – 0.78
Neomycin	3.13	3.13
Spectinomycin	25	12.5
Streptomycin	0.20	0.20
Rifampicin	1.56-3.13	1.56
Meropenem	6.25	6.25
Vancomycin	0.78	0.39
Tunicamycin	5	5
Teicoplanin	12.5-25	12.5
Cycloserine	50	25
Chloramphenicol	25	25
Tetracycline	0.39	0.19
Nalidixic Acid	150	150
Isoniazid	6.25	3.13
Ethambutol	1.56	0.39
Lysozyme	31.25	15.63
Ethidium bromide	8	8

Table S2. Minimum inhibitory concentrations (MIC) of β-lactam compounds tested in the presence and absence of the β-lactamase inhibitor clavulanic acid (CLAV) against *M. smegmatis* WT and Δ*nagA*. MICs were determined as the lowest concentration of drug that prevented the colour change of resazurin (blue –no bacterial growth) to resorufin (pink –bacterial growth) and were performed in triplicate. Where values differed between replicates, a range is stated. The concentration of clavulanic acid is shown as µg/mL,

	**MIC (µg/mL)**	
**Compound**	**WT**	**Δ*nagA***
Ampicillin	100	100
Amoxicillin	12.5	12.5
Piperacillin	> 200	> 200
Ceftriaxone	800	800
Clavulanic acid	400-800	200
Ampicillin + CLAV 100	6.25	1.56
Amoxicillin + CLAV 100	1.56	0.39
Piperacillin + CLAV 100	50	25
Ceftriaxone + CLAV 100	> 200	25-50
Meropenem + CLAV 100	6.25	0.78-1.56

Table S3. Spot assay of compounds tested against *M. smegmatis* WT and Δ*nagA*. WT and Δ*nagA* cells were spotted onto 7H10 agar containing different concentrations of antibiotics for those where differences in MIC values between WT and Δ*nagA* were observed (Table S1). Serial dilutions of cells were spotted, colonies were counted and the percentage recovery of colony forming units (CFU) determined by comparison to a no drug control.

		***M. smegmatis* WT**		***M. smegmatis* Δ*nagA***	
**Compound**	**Concentration**	**CFU/mL**	**Percentage recovery**	**CFU/mL**	**Percentage recovery**
No compound	0 µg/mL	8.3 x10^5	100%	1.6 x10^6	100%
Cycloserine	50 µg/mL	7.7 x10^5	92.8%	1.3 x10^6	81.3%
	30 µg/mL	6.3 x10^5	75.9%	1.3 x10^6	81.3%
	20 µg/mL	1.0 x10^6	100%	1.4 x10^6	87.5%
	10 µg/mL	1.2 x10^6	100%	1.4 x10^6	87.5%
Spectinomycin	25 µg/mL	2.7 x10^5	32.5%	1.7 x10^5	10.6%
	20 µg/mL	3.3 x10^5	39.8%	3.7 x10^5	23.1%
	15 µg/mL	3.7 x10^5	44.6%	5.0 x10^5	31.3%
	10 µg/mL	6.0 x10^5	72.3%	6.3 x10^5	39.4%
Vancomycin	1 µg/mL	1.3 x10^5	15.7%	7.3 x10^4	4.6%
	0.75 µg/mL	3.7 x10^5	44.6%	5.3 x10^5	33.1%
	0.5 µg/mL	5.0 x10^5	60.2%	6.3 x10^5	39.4%
	0.25 µg/mL	9.7 x10^5	100%	1.1 x10^6	68.8%
Tetracycline	0.4 µg/mL	0	0%	0	0%
	0.2 µg/mL	6.7 x10^5	80.7%	8.7 x10^5	54.4%
	0.1 µg/mL	9.0 x10^5	100%	1.3 x10^6	81.3%
	0.05 µg/mL	1.4 x10^6	100%	1.1 x10^6	68.8%
Isoniazid	6 µg/mL	0	0%	0	0%
	4 µg/mL	7.7 x10^5	92.8%	9.7 x10^5	60.6%
	3 µg/mL	9.0 x10^5	100%	1.3 x10^6	81.3%
	2 µg/mL	7.0 x10^5	84.3%	1.4 x10^6	87.5%
Ethambutol	2 µg/mL	0	0%	0	0%
	1 µg/mL	0	0%	0	0%
	0.5 µg/mL	4.0 x10^5	48.2%	1.0 x10^6	62.5%
	0.25 µg/mL	7.0 x10^5	84.3%	1.8 x10^6	100%
Gentamycin	1 µg/mL	8.3 x10^4	10%	2.3 x10^5	14.4%
	0.75 µg/mL	3.7 x10^5	44.6%	3.3 x10^5	20.6%
	0.5 µg/mL	5.0 x10^5	60.2%	8.3 x10^5	51.9%
	0.25 µg/mL	8.0 x10^5	96.4%	9.0 x10^5	56.3%

Table S4. Primers used in this study

Primer name	sequence (5'-3')	Purpose
HL	aggatccaggacctgccaat	Sequencing primers for allelic exchange substrate verification
HR	cttcaccgatccggaggaac	Sequencing primers for allelic exchange substrate verification
OL	cggccgataatacgactca	Sequencing primers for allelic exchange substrate verification
OR	ctgacgctcagtcgaacgaa	Sequencing primers for allelic exchange substrate verification
MSMEG2119_LL	ttttttttcagaaactgtgatcacgcgcaaacccg	Construction of allelic exchange substrate (AES)
MSMEG2119_LR	ttttttttcagttcctggaccagggaggccaccag	Construction of allelic exchange substrate (AES)
MSMEG2119_RL	ttttttttcagagactgtggccgccaccgggatgt	Construction of allelic exchange substrate (AES)
MSMEG2119_RR	ttttttttcagcttctgcgtccaggcgcaccagca	Construction of allelic exchange substrate (AES)
msnagA-3F	gttcaccacccacgtcgc	RT-PCR crr
msnagA-3R2	gacgcggtgatgtttccc	RT-PCR crr
msnagA-4F	ctgcctcacccagggcct	RT-PCR nagB
msnagA-4R2	cgtcgagcaacacggtcacg	RT-PCR nagB
msnagA-5F2	cgtggttgtcgaccctgcgg	RT-PCR nagA
msnagA-5R2	gccgttccggcccaacggtc	RT-PCR nagA
mysA_F	cgaggacgaggaagaagaag	RT-PCR mysA
mysA_R	ttgtagcccttggtgtagtcg	RT-PCR mysA

Experimental procedures

RNA isolation and operon analysis

M. smegmatis strains were grown to late-log phase (OD_{600} = 0.8-1) before harvesting and snap-freezing the pellets in liquid nitrogen. The pellets were stored at -80 ºC before RNA isolation. RNA was isolated using the Total RNA extraction kit (Monarch). The pellets were resuspended in 800 mL 1x DNA/RNA protection reagent (Monarch) and processed with bead beating (FastPrep® Lysing Matrix B tubes (FastPrep-24, MP Biomedics)). Total RNA was isolated according to the 'tough-to-lyse sample' protocol of the Total RNA extraction kit (Monarch), treated with Turbo DNase (Turbo DNA-free kit, Ambion) to remove contaminating DNA. RNA was confirmed free of contaminating gDNA by PCR of *mysA* with the primers in Table S2, and synthesis of cDNA was performed using the first strand cDNA synthesis kit (APExBIO, UK) with random hexamer priming according to the manufacturer's instructions. The isolated RNA and generated cDNA were stored at −80 °C. Regions were amplified from cDNA using Q5 DNA polymerase (NEB, UK) with the primer pairs indicated in Table S2 (msnagA-3F, msnagA-3R2, msnagA-4F, msnagA-4R2, msnagA-5F2, msnagA-5R2, mysA_F, mysA_R) and analysed on 0.8% agarose gel. A negative control of RNA and positive control using *M. smegmatis* gDNA were also included.

Lipid analysis

Cultures were grown to OD_{600} = 0.8 in 7H9 broth, pelleted by centrifugation (3,220 × *g*, 10 min, room temperature), washed with PBS and then dried at 55 °C under air flow. The apolar and polar lipid fractions were isolated as described previously (1). Briefly, apolar lipids were extracted from the pellet by the addition of methanol/0.3% NaCl (10:1 v/v) (2 mL) and petroleum ether 60-80 (2 mL). The polar lipids were then extracted from the lower organic phase (methanol/0.3% NaCl (10:1 v/v)) by the addition of chloroform/methanol/0.3% NaCl (9:10:3 v/v) (2.3 mL) before centrifugation (3,220 × *g*, 10 min, room temperature) and removal of the supernatant. The remaining pellet was further extracted chloroform/methanol/0.3% NaCl (5:10:4 v/v) (750 µL) before centrifugation (3,220 × *g*, 10 min, room temperature) and removal of the supernatant. Fatty acid methyl esters (FAMES) and mycolic acid methyl esters (MAMES) were extracted by incubating the delipidated pellet in 5% tetrabutylammonium hydroxide (2 mL) at 95 °C for 16 h. Water (1 mL) iodomethane (50 µL) and dichloromethane (2 mL) were added, and the organic phase subjected to repeated washing with water before evaporating to dryness. The resultant residue was resuspended in diethyl ether and sonicated (sonicating water bath (Ultrawave), 10 min, room temperature) to yield the FAMEs and MAMEs. The extracted FAMES and MAMES were evaporated to dryness at 70 °C and resuspended in chloroform/methanol (2:1 v/v) (100 µL).

The lipid extracts were resolved by TLC using the following solvent systems. System A: direction 1: petroleum ether 60-80/ ethyl acetate (98:2) 3x, direction 2; petroleum ether 60-80/acetone (98:2); 1x System B: direction 1: petroleum ether 60-80/acetone (92:8) 3x, direction 2: toluene/ acetone (95:5) 1x; System C: direction 1: chloroform methanol (96:4) 1x, direction 2: toluene/acetone (80:20) 1x; System D: direction 1:

chloroform/methanol/water (100:14:0.8) 1x, direction 2: chloroform/methanol/water (60:30:6) 1x, direction 2: chloroform/ acetone/ methanol/ water (50:60:2.5:3) 1x; System E: direction 1: chloroform/ methanol/water (60:30:6) 1x, direction 2: chloroform/ acetic acid (glacial)/methanol/water (40:25:3:6) 1x and System F: petroleum-ether 60-80/ acetone (19:1) 3x. TLCs were visualised using 10% phosphomolybdic acid in ethanol.

References

1. Besra GS. 1998. Preparation of cell-wall fractions from mycobacteria. Methods Mol Biol 101:91-107.
